# LRP2 is a potential molecular target for nonsyndromic pathological myopia

**DOI:** 10.1172/jci.insight.192929

**Published:** 2025-06-24

**Authors:** Kimberley Delaunay, Emilie Picard, Patricia Lassiaz, Laurent Jonet, Vidjea Cannaya, José Maria Ruiz-Moreno, Kentaro Kojima, Henrik Vorum, Bent Honoré, Jorge Ruiz-Medrano, Lasse Jørgensen Cehofski, Eric Pussard, Renata Kozyraki, Alicia Torriglia, Olivier Cases, Francine Behar-Cohen

**Affiliations:** 1 Centre de Recherche des Cordeliers, Sorbonne Université, INSERM, Université de Paris Cité, Paris F-75006, France.; 2 Servicio de Oftalmologia, Hospital universitario Puerta de Hierro-Majahonda, Universidad Castilla La Mancha, Albacete, Spain.; 3 Department of Ophthalmology, Kyoto Prefectural University of Medicine, Kyoto, Japan.; 4 Department of Ophthalmology, and; 5 Department of Clinical Medicine, Aalborg University Hospital, Aalborg, Denmark.; 6 Department of Biomedicine, Aarhus University, Aarhus, Denmark.; 7 Department of Genetic and Hormonology, Assistance Publique-Hôpitaux de Paris, Hôpital du Kremlin Bicêtre, Université de Paris-Saclay, Kremlin-Bicetre F-94270, France.; 8 INSERM U1096, Faculty of Health, University of Rouen, Rouen F-76000, France.; 9 Assistance Publique-Hôpitaux de Paris, Hôpital Cochin Ophtalmopole, Paris F-75014, France.; 10 Hôpital Foch, Department of Ophthalmology, Suresnes, France.

**Keywords:** Neuroscience, Ophthalmology, Retinopathy

## Abstract

High myopia (HM) and posterior staphyloma (PS) are major causes of vision loss worldwide. Genetic and environmental factors, especially light exposure, influence myopia. This study shows that low-density lipoprotein–related receptor type 2 (LRP2) levels are decreased in the vitreous of patients with HM and PS, and that in human donor eyes affected by PS, LRP2 expression was reduced in the neural retina and retinal pigment epithelium (RPE), with morphologic changes similar to those observed in the *Foxg1-Cre*-*Lrp2^fl/fl^* mouse that also develops PS. In human induced pluripotent stem cell–derived RPE cells, LRP2 silencing regulated genes involved in eye and neuronal development, visual perception, tissue remodeling, hormone metabolism, and RPE structure. Its expression increased under light exposure, particularly red light, but was downregulated by cortisol. These findings establish a link between LRP2, myopization, and environmental factors, highlighting its crucial role in nonsyndromic HM and PS. LRP2 appears to be a promising therapeutic target for HM treatment.

## Abstract 2

LRP2 plays a pivotal role in the pathophysiology of high myopia and posterior staphyloma, and its expression in retinal pigment epithelium is influenced by light exposure and cortisol levels.

## Graphical abstract

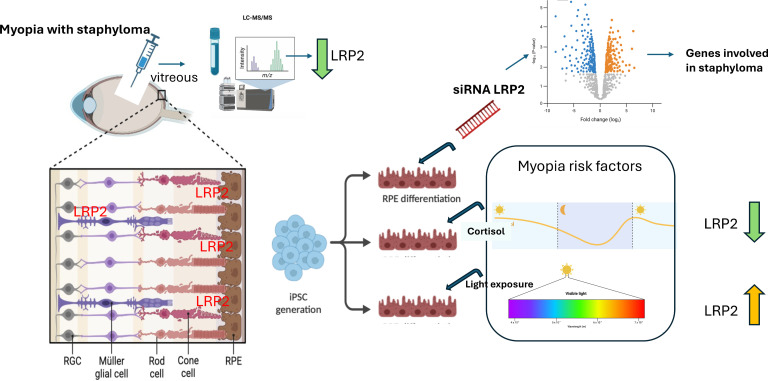

## Introduction

The prevalence of myopia, a refractive error commonly caused by abnormal elongation of the eyeball, is increasing rapidly worldwide, with the global prevalence expected to exceed 50% by 2050 (1). The refractive error can be corrected by optical or surgical means, but high myopia (HM), greater than 6 diopters, is often associated with sight-threatening retinal complications, including retinal detachment and maculopathy secondary to posterior staphyloma (PS). PS is a focal protrusion of the ocular globe with an extreme thinning of the retinal pigment epithelium (RPE), the choroid, the neural retina, and the sclera (2, 3). The prevalence of PS is estimated around 70% in eyes with HM (4). To date, there is no method to prevent HM and PS and its blinding complications, which is a major public health problem (5).

Myopia results from a complex interplay between genetic and environmental factors (6, 7), the most widely accepted of which is exposure to daylight, to the point where light therapy has been proposed as a preventive strategy. However, the respective effects of different light components (such as luminance, spectral composition, temporal modulation) and of circadian regulations (8–10) remain incompletely understood. Several hundreds of genes, most of which are involved in TGF-β signaling, collagen synthesis, and retinal signal transduction have been associated with myopia (11) and the study of monogenic syndromic myopia can help understanding of the mechanisms involved in multifactorial myopia. Variants in the gene encoding low-density lipoprotein (LDL) receptor–related protein 2 (*LRP2*) cause the rare Donnai-Barrow syndrome (DBS, OMIM 222448), which combines pathologic myopia and PS, hypertelorism, craniofacial malformations, sensorineural hearing loss (SHL), agenesis of the corpus callosum (ACC), and proteinuria (12–14). Twenty-three different *LRP2* variants are associated with DBS and most are loss-of-function alleles. A recent study describes an association of pathogenic variants of *LRP2* causing only severe myopia with PS and SHL (15), suggesting that one of the two *LRP2* variants likely retains partial function and that a wide spectrum of myopic phenotypes, including PS or retinal detachment, could result from various levels of LRP2 function.

Furthermore, mouse models with conditional knockout of *Lrp2* (*Lrp2*-cKO) in all retinal tissues (*Foxg1-Cre*-*Lrp2^fl/fl^*) develop early postnatal severe myopia and a protrusion of the eye globe very similar to PS (16). Conditional mutants in which *Lrp2* was knocked out only in the RPE also developed myopia and buphthalmos (17), demonstrating the important role played by LRP2 in the RPE in driving the myopia phenotype. In addition, transient postnatal *Lrp2* downregulation in the RPE promoted eye growth, and high *Lrp2* expression in the RPE ensured that eye growth stopped at the correct size (18). Taken together, these findings highlight the key role played by LRP2 in the RPE in the control of eye growth.

LRP2, known as megalin, is a large transmembrane glycoprotein detected at the apical membrane of various epithelial cells where it participates in clathrin-mediated endocytosis of ligands that are either degraded in lysosomes or transported to the basal pole of the cell, where they are secreted (19). It regulates the extracellular concentrations of molecules, including vitamins and hormones (20, 21), and modulates the activity of morphogens such as sonic hedgehog (SHH) or bone morphogenetic protein 4 (BMP4) during development (22, 23). Via endosome recycling, LRP2 also controls cell shape and planar cell polarity (24, 25). The availability of LRP2 at the cell surface is subject to regulatory mechanisms, including phosphorylation and the shedding of an ectodomain (26). In murine RPE, LRP2 is predominantly located at the apical plasma membrane, where it facilitates the uptake of albumin or transferrin (20). How LRP2 expressed in the RPE controls eye growth is unknown. Whether LRP2 is involved in nonsyndromic HM (NSHM) and in PS in humans is unknown.

## Results

### Levels of LRP2 are decreased in the vitreous of NSHM eyes with PS.

LRP2 levels were evaluated in vitreous samples of patients with HM and PS who underwent pars plana vitrectomy for myopic tractional maculopathy, macular hole, or epiretinal membrane and compared to vitreous from emmetropic patients operated for epiretinal membrane or for intraocular lens luxation. These measurements were carried out on a Japanese population and on a White European population by 2 independent research groups and 2 different analytical methods. Table 1 recapitulates the demographic characteristics and surgical indications.

**Figure 1:**
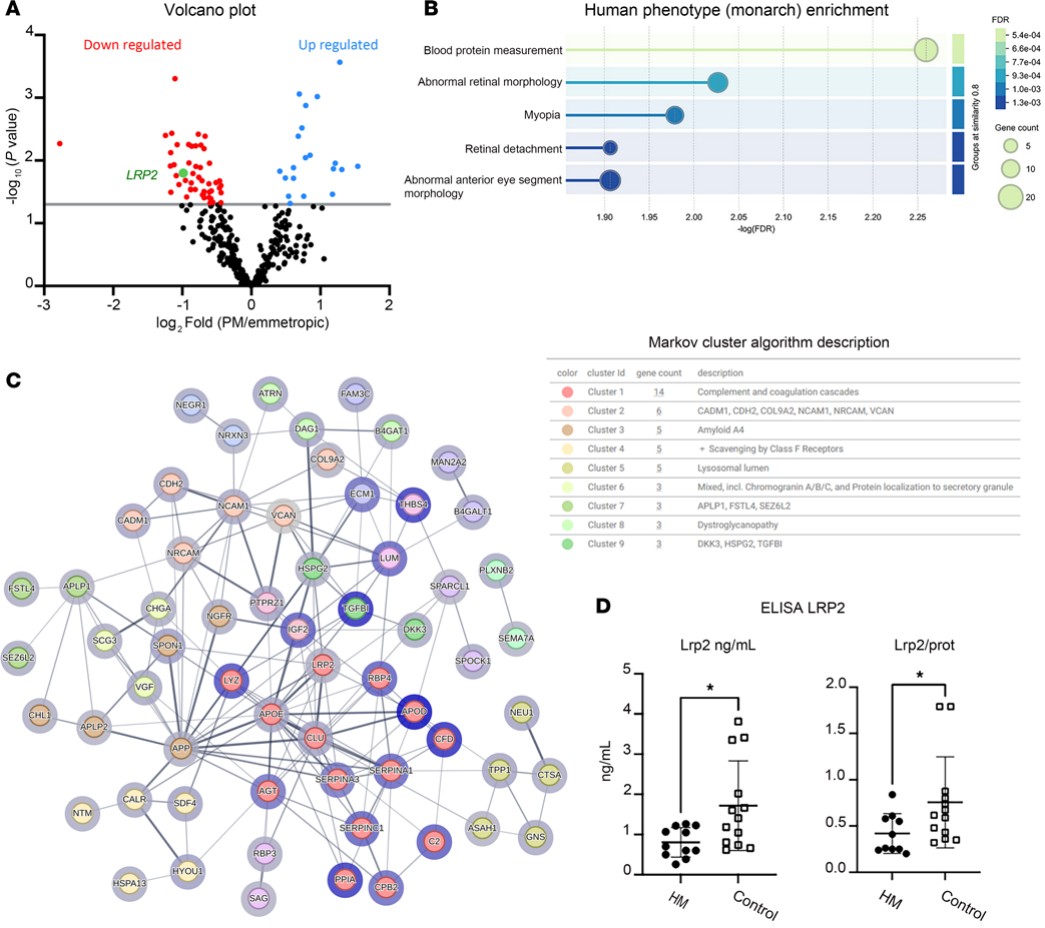
Analysis of proteins in the vitreous of myopic and emmetropic patients. (**A**) Volcano plot of differentially expressed proteins. The rightmost part of the plot (blue circles) shows the 20 upregulated proteins and the leftmost (red circles) the 53 downregulated proteins. PM, pathological myopia. (**B**) Monarch analysis (https://monarchinitiative.org/) enrichment of downregulated proteins; only the top 5 terms are displayed. Gene count associated with a particular Monarch term is indicated. (**C**) Protein-protein interactions (PPIs) using STRING analysis (see Supplemental Methods), displaying the signaling network between the differentially expressed proteins. Nodes in blue circles refer to upregulated proteins, nodes in gray circles refer to downregulated proteins. Markov cluster algorithm analysis indicated the main interactomes. The 9 top interactomes are indicated at the right. (**D**) Concentrations of LRP2 protein in ng/mL in undiluted vitreous from control (*n* = 13) and NSHM (*n* = 10) eyes quantified with ELISA. The graph on the right shows the ratio of LRP2 compared to the total protein content. Statistical analysis was performed using the Mann-Whitney *U* test. *P < 0.05.

A proteomic analysis was performed on the vitreous from 15 Japanese patients with NSHM compared to 10 controls. Axial length in the HM group was 29.33 ± 1.94 mm and all patients had PS. Proteomic analysis of the vitreous performed by label-free quantitative liquid chromatography–tandem mass spectrometry identified 332 proteins (Supplemental Data File 1; supplemental material available online with this article; https://doi.org/10.1172/jci.insight.192929DS1), of which were 73 differentially expressed proteins (DEPs), including LRP2 (fold change = 0.5; *P* = 0.016) (Figure 1A and Tables 2 and 3). The top upregulated DEPs included apolipoprotein D, TGF-β–induced ig-H3 (TGFBI), complement factor D, and thrombospondin 4. On the other hand, versican core protein, S-arrestin, collagen IX α2 chain, LRP2, and retinol-binding protein 3 (IRBP) were reduced. The most significant biological processes associated with downregulated proteins were linked to nervous system development and negative regulation of proteolysis (Supplemental Figure 1). Molecular functions related to the significantly regulated proteins included reduced LDL binding, calcium ion binding, and chaperone binding, whereas serine-type endopeptidase inhibitor activity and integrin binding were elevated (Supplemental Figure 1). The downregulated proteins were predominantly located in the extracellular region and extracellular space, while upregulated proteins were mostly linked to extracellular matrix (ECM) (Supplemental Figure 1). Abnormal retinal morphology, myopia, and retinal detachment were the most significant phenotypes associated with the DEPs (Figure 1B). The most significant connected protein cluster contained 14 proteins, including LRP2 and its ligands clusterin (CLU) and apolipoprotein E (APOE) (Figure 1C and Supplemental Figure 2A), which were enriched in neurodegenerative disorders (Supplemental Figure 2B). In the White European group of patients, 10 HM patients and 13 emmetropic patients were included. In the HM group, axial length was 29.67 ± 2 mm and all eyes had PS. LRP2 was measured by enzyme-linked immunosorbent assay (ELISA) analysis on vitreous samples. Total protein–normalized LRP2 levels were significantly lower in HM eyes (0.8 ± 0.36 and 0.41 ± 0.21 ng/mL) than in emmetropic eyes (1.7 ± 1.1 and 0.75 ± 0.49 ng/mL) (*P* = 0.01 and 0.02, respectively) (Figure 1D).

### Pathological features of HM human eyes with PS.

**Figure 2:**
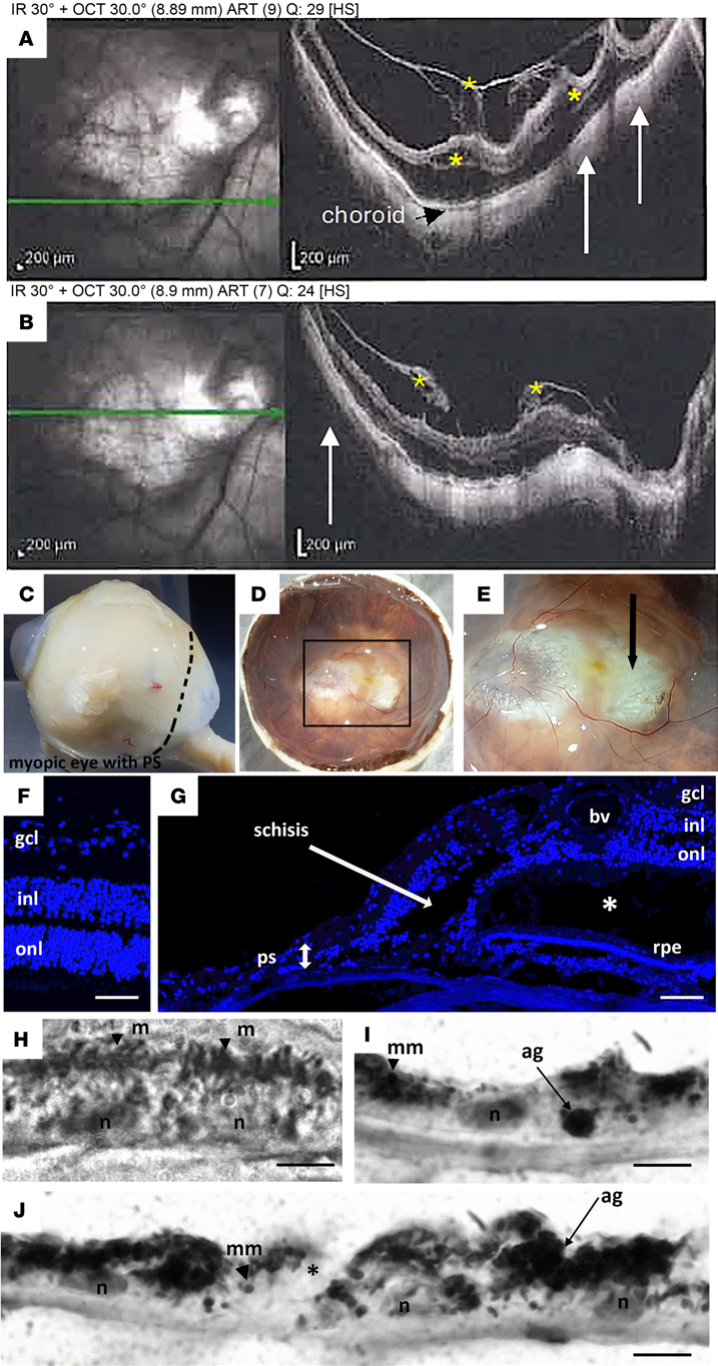
Ophthalmologic imaging and histological analysis of the right eye of an HM donor. (**A** and **B**) Premortem SD-OCT scans of the right eye with inferior cross section (**A**) and macular cross section (**B**), showing the posterior staphyloma (PS) (white arrows) and tractional maculopathy (yellow stars). The myopic eye displayed an atrophy of the macula delineated by small arrows and the posterior incurvation indicated the uncomplicated PS. (**C**) Gross morphology of the HM right enucleated postmortem eye with PS. (**D**) Macroscopic image of the posterior chamber after dissection showing the PS. (**E**) Insert box of **D** focusing on fovea and PS (black arrow). (**F**) DAPI-stained section of a normal human retina near the macula. (**G**) DAPI-stained section of the HM retina with a PS (double headed arrow) shows a severe reduction in thickness of all retinal layers, adjacent to the PS. Schisis (white arrow) is observed between the inner nuclear layer (inl) and the outer nuclear layer (onl). A cyst is observed between the RPE and the outer segments layer (asterisk). bv, blood vessel. (**H**) RPE cells in human retina form a single layer of pigmented cells as observed on light transmission microscopy. Melanosomes (m) have an elongated shape and nuclei (n) are spaced at regular intervals. (**I** and **J**) In the HM donor eye, RPE cells are still forming a single layer. RPE cells are abnormally large, their apical domain is reduced, and melanosomes aggregate in macromelanosomes (mm) or macrostructures (ag). Scale bars: 75 μm (**F**), 120 μm (**G**), and 5 μm (**H**–**J**).

**Figure 3:**
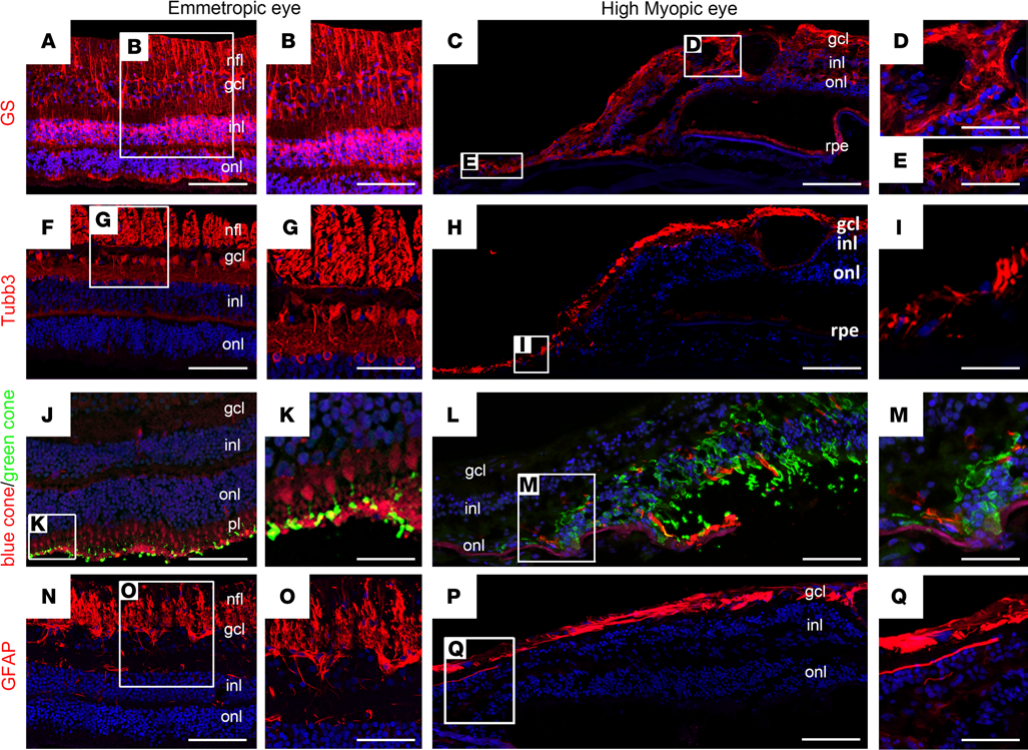
Immunolabeling of retinal cells in the emmetropic and HM retina. (**A** and **B**) Glutamine synthetase (GS) labels Müller glial cells in the emmetropic retina, extending radially from the nerve fiber layer (nfl) to the outer nuclear layer (onl). (**C** and **D**) In HM retina, GS-positive cells extend in all directions and thicken at the retinal surface. At higher magnification, GS-positive cells surround a cyst in the ganglion cell layer (gcl) (**C**) and fill the bottom of the staphyloma where almost no retina remains (**E**). (**F** and **G**) Tubulin β3 (Tubb3) is a neuronal marker of retinal ganglion (RGC) and amacrine cells in the emmetropic retina. (**H**) In HM retina, the number of Tubb3-positive cells and RGCs is reduced. Higher magnification shows Tubb3-positive axons browsing at the surface of the staphyloma. (**J** and **K**) Blue (red staining) and green (green staining) opsin markers reveal the distribution of blue and green cones in the emmetropic retina. (**L** and **M**) In HM retina, the number of blue cones is reduced (**L**) and high magnification shows the accumulation of green opsin in abnormally shaped outer segments. (**N** and **O**) Glial fibrillary acidic protein (GFAP) labels astrocytes and glial Müller cell endfeet in the emmetropic retina. (**P** and **Q**) In the HM retina, the GFAP-positive endfeet are reduced. (**Q**) At higher magnification, a GFAP-positive membrane lays on the retinal surface but no positive cells are observed in the retina. Scale bars: 200 μm (**A**, **F**, **J**, and **N**), 80 μm (**B**, **G**, and **O**), 20 μm (**K**), 250 μm (**C**, **H**, **L**, and **P**), and 60 μm (**D**, **E**, **I**, **M**, and **Q**).

Two HM postmortem eyes, with axial lengths of 32 mm and 31 mm for the right and left eye, and a PS, were studied. The premortem spectral domain optical coherence tomography (SD-OCT) images of the right pseudophakic HM eye revealed posterior incurvation of the PS with thinning of the retina and the choroid (Figure 2, A and B; black arrows), complicated by myopic tractional maculopathy (MTM) with foveoschisis (Figure 2, A and B; stars and white stars). The SD-OCT images of the left eye were of low quality due to corneal opacification. On the postmortem enucleated donor eyeball, the PS with scleral thinning was clearly visible (Figure 2C). Peripapillary atrophy was visible on macroscopic pictures of the open eyeball (Figure 2, D and E), and the PS was located temporal to the fovea (Figure 2, D and E; black arrow). A cross section at the level of the PS (Figure 2G) showed disorganization of the retinal layers and cysts (arrows) and extreme retinal thinning at the bottom of the PS (Figure 2G; double arrow) compared with emmetropic retina (Figure 2F). RPE cells outside the PS showed clear disorganization (Figure 2, H–J), with mislocalization of melanosomes and aggregations of pigments. More precise cellular disorders were characterized using markers of different cell types on immunohistochemistry of emmetropic and HM human retina cryosections (Figure 3). In the control retina, glial Müller (RMG) cells identified by glutamine synthetase (GS) expression extended their processes from the inner limiting membrane to the outer limiting membrane (Figure 3, A and B). In the HM retina, RMG processes were disorganized and lost their alignments within the thinned layers (Figure 3C). The RMG processes organized around cysts (Figure 3D) and invaded the bottom of the PS that was covered by RMG cells (Figure 3E). The neuron-specific marker tubulin β3 labeled the nerve fiber layer, the ganglion cells (RGCs) and their extensions, and amacrine cells (Figure 3, F and G). In the HM retina, a severe thinning of the nerve fiber layer (Figure 3H) was observed together with a reduction in RGCs (Figure 3, H and I). Blue and green cones (Figure 3, J and K), labeled by opsins, showed significant disorganization in HM retina with greater loss of blue cones (Figure 3, L and M). Finally, the astrocyte-specific marker glial fibrillary acidic protein (GFAP), which stains astrocytes and the end feet of RMG cells in the emmetropic retina (Figure 3, N and O), showed a dense layer at the surface of HM retinas, which could correspond to the vitreoretinal tractional membrane seen on SD-OCT (Figure 2, A and B) but the RMG invading the PS did not express GFAP. The retina of the HM eye exhibited loss of RGCs and cones, as well as thinning of all retinal layers, and cystic degeneration accompanied by RMG cell disorganization. Extreme degeneration was present in the PS area, which was filled by RMG cells.

### Decreased LRP2 in human HM with PS retina is associated with severe RPE disorganization similar to Foxg1-Cre-Lrp2^fl/fl^ mouse RPE.

**Figure 4:**
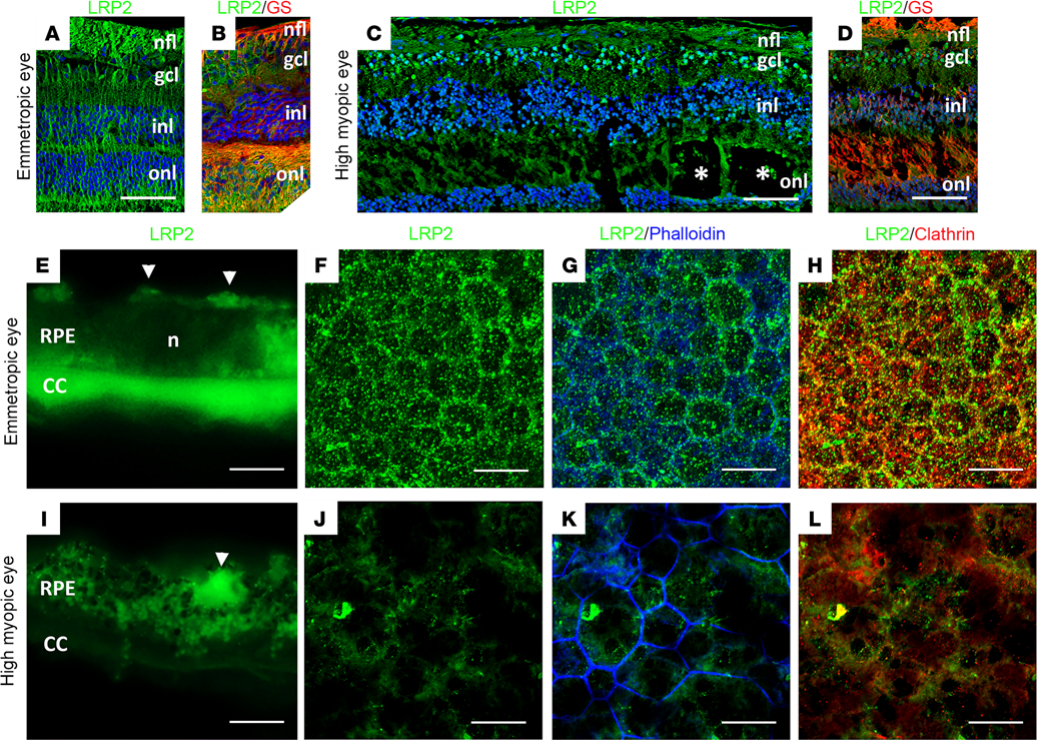
LRP2 immunolabeling in the emmetropic and HM retina. (**A** and **B**) In the emmetropic neural retina, LRP2 is expressed around RGCs and along GS-positive glial Müller cells. (**C** and **D**) In the HM neural retina, LRP2 expression is greatly reduced in GS-positive cells, which surround cysts (stars). (**E**) In transversal sections of RPE cells, LRP2 is located at the apical pole (white arrowheads), in intracellular vesicles, and at the basal pole of the RPE, and in the pillars of the choriocapillaris. (**F** and **G**) In an RPE flat-mounted preparation from the left emmetropic eye, LRP2 is distributed in cytoplasmic vesicles (**F**) along apical and lateral membranes (**G**) and most LRP2-positive vesicles are also positive for clathrin. (**I**) In transversal section of the HM RPE, LRP2 expression is greatly diminished and absent in the choriocapillaris. (**J**–**L**) In RPE flat mounted from the HM eye, LRP2 distribution is sparse and diffuse, and does not colocalize with clathrin (**L**) that is also diminished. Scale bars: 200 μm (**A**–**D**), 10 μm (**E** and **I**), and 50 μm (**F**, **G**, and **J**–**L**).

**Figure 5:**
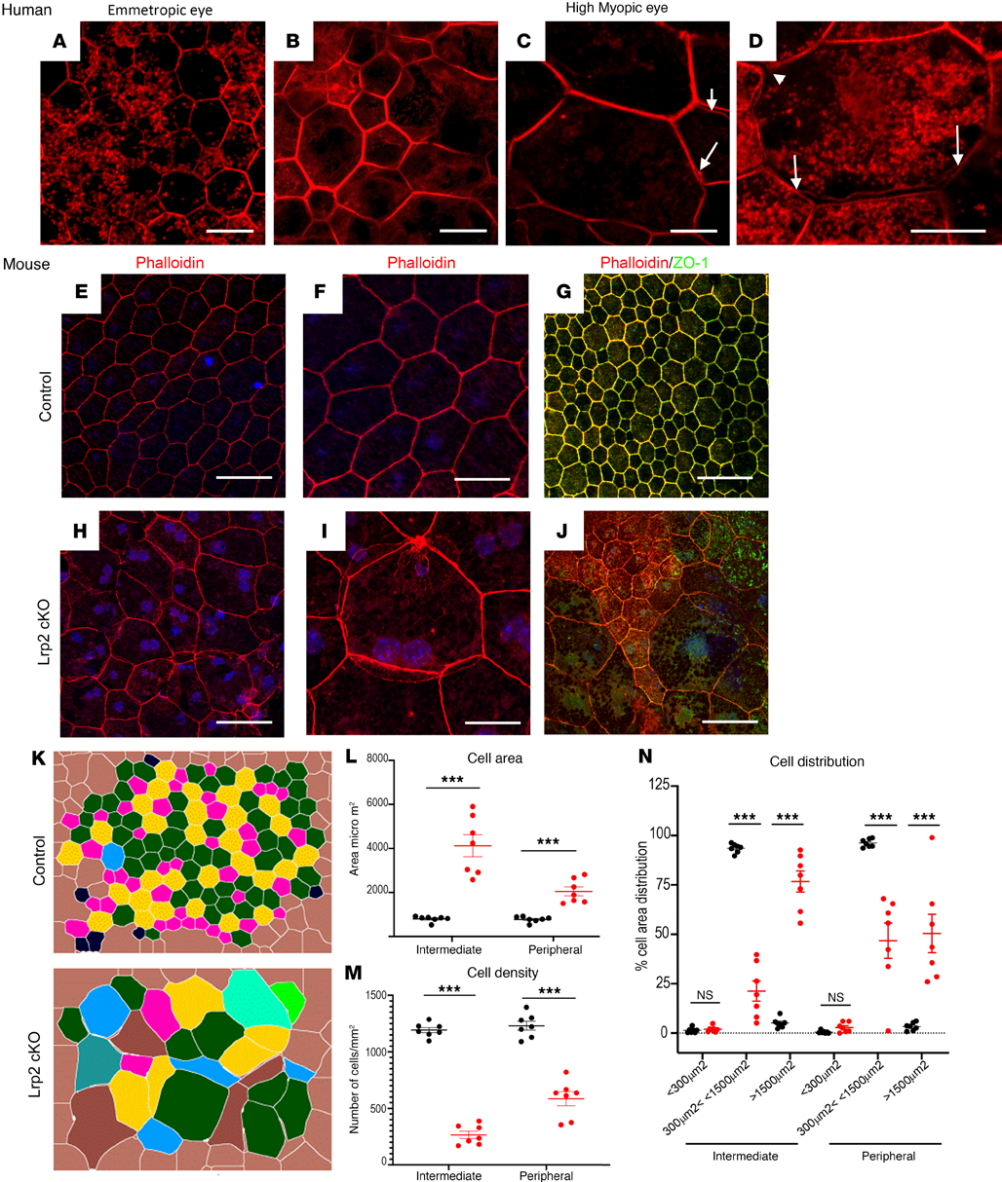
Organization of the RPE is altered in HM eye, similar to the RPE in *Lrp2*-cKO mice. (**A**) Phalloidin staining reveals the geometric paving pattern of RPE in the emmetropic eye. (**B**–**D**) In HM eye, RPE geometric paving pattern is lost as most cells increase in size. Bicellular junctions display secondary actin arcs (**C**, arrows) and cell junctions are disorganized (**D** arrows). (**E** and **F**) Phalloidin staining on RPE flat mount of WT control mouse shows the regular pattern of RPE cells. (**G**) Zonula occludens 1 (ZO-1) follows the distribution of phalloidin in the apical pole. (**H** and **I**) In *Lrp2*-cKO RPE, the pseudogeometric paving pattern is lost and is replaced by a tangle of cells whose surface has increased. (**J**) ZO-1 is redistributed in the cytoplasm of abnormally shaped RPE cells. (**K**) Digital overlay reconstruction of control and *Lrp2*-cKO RPE indicates the increase size in cells both at the periphery and at the intermediate level of the retina in *Lrp2*-cKO RPE. (**L**) Cell area in μm^2^, (**M**) cell density in number of cells/mm^2^, and (**N**) distribution of cells by sizes in the intermediate and peripheral retina of control compared to *Lrp2*-cKO mice. Values represent the mean of cell size average of each sample (*n* = 7) per genotype ± SEM. Mann-Whitney *U* test. ***P < 0.001. Scale bars: 50 μm (**A**–**D**, **E**, and **H**), 25 μm (**F** and **I**), and 75 μm (**G** and **J**).

In emmetropic human neural retinas, LRP2 was expressed in RMG cells, colocalized with GS (Figure 4, A and B). In the HM neural retinas, significant disorganization of the retinal layers was evident at the margin of the staphyloma, where RMG cells spanning through layers and the subretinal space expressed low levels of LRP2 (Figure 4, C and D). In the RPE cells, LRP2 expression was decreased in the HM eye, as seen in both transversal sections from the left eye (Figure 4E) and flat mounts of the right eye (Figure 4, F–H). In emmetropic RPE, LRP2 was located at the apical membrane and in vesicles (Figure 4E; arrowhead) but also showed diffuse labeling underneath Bruch’s membrane, in the most inner part of the choroid where it stained the pillars of the choriocapillaris (Figure 4E). In RPE flat mounts of the emmetropic control eye, LRP2 was localized in punctiform submembrane clusters at the apical and lateral borders of the RPE cells (Figure 4, F and G), at the cell membrane (Figure 4G), and in clathrin-positive vesicles (Figure 4H). On transversal sections of HM RPE cells (Figure 4I), LRP2 was faintly detected (Figure 4, J–L) at the cell membrane (Figure 4K) and in very few clathrin-positive vesicles (Figure 4L), with staining of LRP2 aggregated at the apical side (Figure 4I; arrowhead) without any signal underneath Bruch’s membrane (Figure 4I). Along with LRP2 reduction, a parallel reduction in clathrin-positive vesicles (Figure 4L) was observed. In HM eyes with PS, LRP2 was thus decreased in the vitreous, in retina, and in the RPE. As opposed to the regular arrangement of RPE cells in emmetropic human eye (Figure 5A), in HM eyes, the RPE displayed irregular organization, cell size dispersity (Figure 5B), and doubling or breaks of the cell junctions (Figure 5, C and D). The RPE from *Foxg1-Cre*-*Lrp2^fl/fl^* mice was studied to explore the impact of suppressed LRP2 expression on the RPE morphology. As opposed to control WT mice (Figure 5, E–G) that showed each regular octagonal RPE cell surrounded by 8 regular hexagonal cells, the transgenic mouse displayed an irregularly arranged RPE with dramatic changes in cellular size and shape mostly posterior to the equator (Figure 5, H and I). Membrane ZO-1 staining in tight junctions was lost and replaced by diffuse cytoplasmic staining (Figure 5J). At the periphery, areas with normal-sized RPE cells showed actin stress fibers (Figure 5J). Automatic quantification (Figure 5K) revealed that RPE cell size increased in the middle and peripheral retina (Figure 5, L and M) and that cell density decreased mostly posterior to the equator (Figure 5N).

The RPE from individuals with HM and PS, which exhibited a marked decrease in LRP2 expression, displayed morphological changes analogous to those observed in *Lrp2*-cKO mice.

### Transcriptional consequences of LRP2 silencing in iRPE cells.

**Figure 6:**
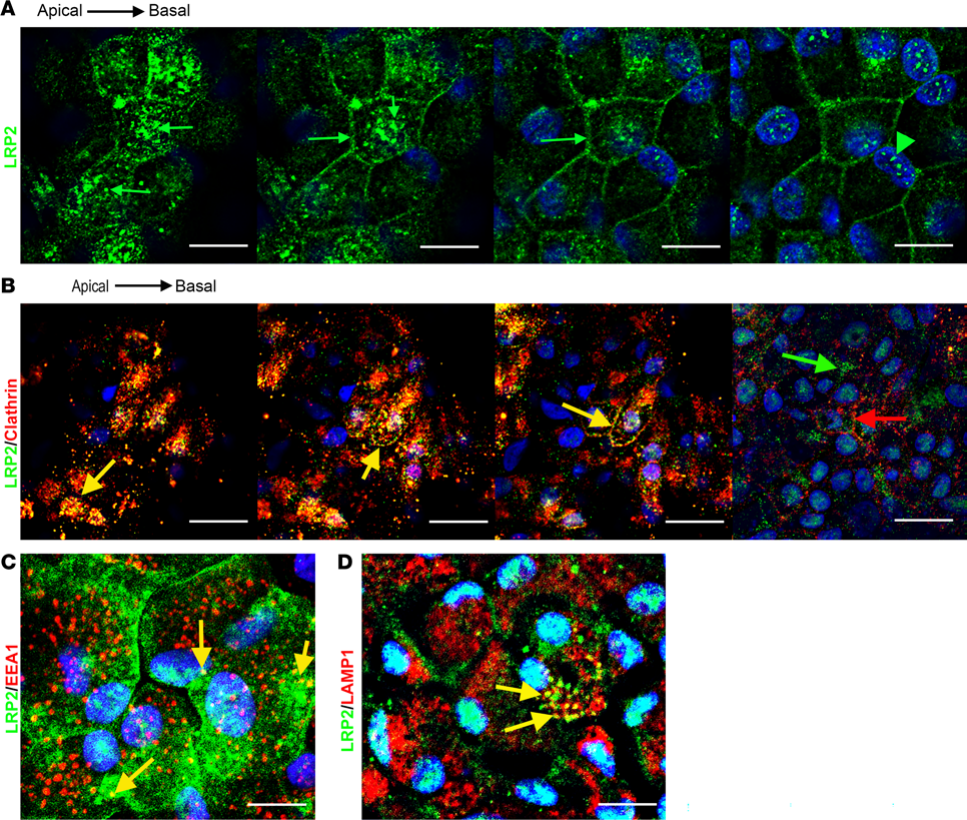
LRP2 localization in healthy human iRPE. (**A**) Consecutive confocal images from an apical to basal stack showing LRP2 accumulation in large vesicular structures in the apical pole (arrows), at lateral membranes, in small cytoplasmic vesicles, and in larger perinuclear vesicles (arrowheads). (**B**) Consecutive confocal images from an apical to basal stack show that LRP2 colocalized with the major protein of coated pits and apical endocytic vesicles, clathrin (yellow arrow). In the basal part of the cell, LRP2 (green arrow) did not colocalize with clathrin (red arrow). (**C**) Confocal image showing that LRP2 (green) partially colocalized with the endocytic marker, early endosome antigen 1 (EEA1; red) (yellow arrow). (**D**) In the basal compartment, LRP2 (green) colocalized with LAMP1 (red), a specific lysosomal marker (yellow arrows). Scale bars: 10 μm (**A**, **C**, and **D**) and 14 μm (**B**).

**Figure 7:**
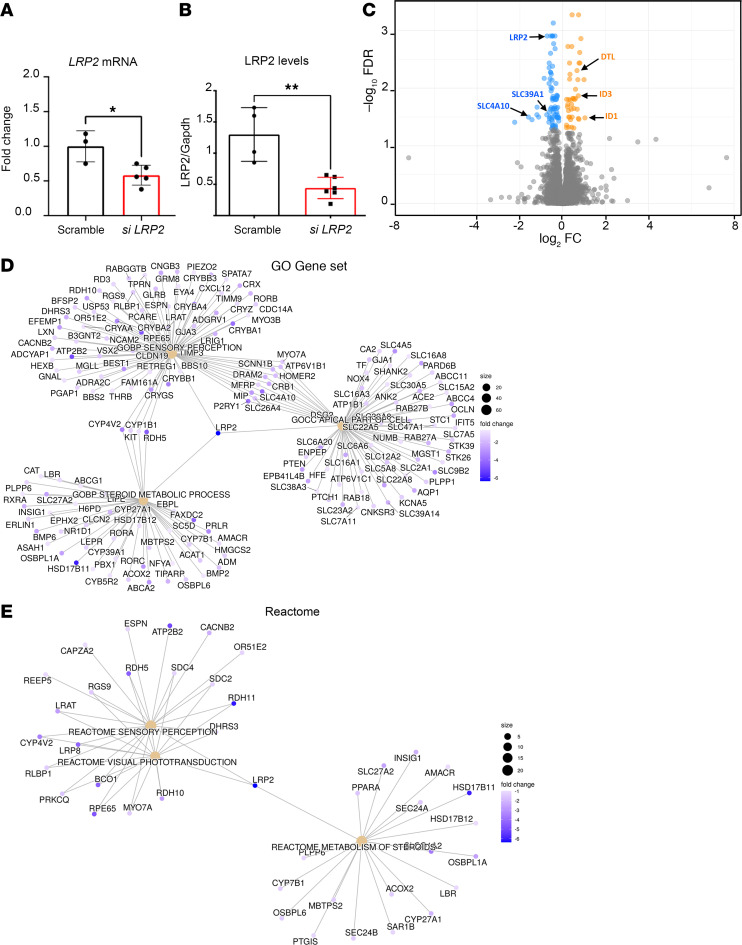
Differentially expressed genes (DEGs) between *siLRP2* and scrambled iRPE cells. (**A**) *Lrp2* mRNA quantification between scrambled (*n* = 4) and *siLRP2* (*n* = 4) iRPE (*P < 0.05). (**B**) LRP2 levels as expressed by the ratio LRP2/GAPDH between scrambled (*n* = 4) and *siLRP2* (*n* = 4) iRPE (**P < 0.01). (**C**) Volcano plot of DEGs between *siLRP2* and scrambled iRPE cells with –log_10_ of the adjusted *P* value on the *y* axis and log_2_ of the fold change in expression on the *x* axis. Some genes, including LRP2, are indicated. (**D**) Category netplot gene enrichment analysis considering only LRP2 as a common factor with 3 GO terms (sensory perception, import across plasma membrane, and steroid metabolic processes) and (**E**) with 2 Reactome terms (sensory perception, visual transduction and metabolism). Fold change (color codes on each graph) for each gene of the selected GO term is indicated. Analysis was performed using Metascape (https://metascape.org/). Absolute normalized enrichment allowed identifying terms that were upregulated or downregulated using all DEGs.

To decipher the consequence of LRP2 downregulation, we used RNA interference to silence *LRP2* mRNA (*siLRP2*) in human induced pluripotent stem cell–derived RPE (iRPE) cells. We first evaluated the ability of differentiated iRPE to express LRP2 and other endocytosis markers. As expected, LRP2 was localized at the cell membrane from the apical to the basolateral side and in subapical vesicles (Figure 6A), colocalizing at the apical pole with the receptor-mediated endocytic protein clathrin (Figure 6B), partially with the early endocytic marker EEA1, (Figure 6C) and with LAMP1, a membrane protein principally located in lysosomes (Figure 6D). Bulk transcriptomes showed that *siLRP2* reduced *LRP2* mRNA expression by 40% after 48 hours, which was confirmed by quantitative PCR (qPCR) (Figure 7A) and by Western blot (Figure 7B). Principal component analysis (PCA) showed the overall variation among samples, with a clear separation in the first 3 components between *siLRP2*-treated and scrambled siRNA–treated iRPE cells (Supplemental Figure 3). We identified 118 differentially expressed transcripts between *siLRP2* iRPE and scramble iRPE, including 75 downregulated and 42 upregulated transcripts (Figure 7C and Supplemental Data File 2). Shortened lists of differentially expressed genes (DEGs) with the greatest fold change are provided in Tables 4 and 5. Complete lists of DEGs with additional information about their roles in eye physiology are provided in Supplemental Tables 1 and 2. Along with *LRP2*, we identified several other RPE-specific apical membrane DEGs that were downregulated in *siLRP2* iRPE, while upregulated DEGs were involved in the control of the circadian clock, cell cycle, and in the regulation of TGF-β activity. The results of the Kyoto Encyclopedia of Genes and Genomes (KEGG) enrichment analysis demonstrated that DEGs were mainly enriched in “retinol metabolism” (downregulation) and in “ECM receptor interaction” (upregulation) (Supplemental Figure 4A and Supplemental Data File 3). The Gene Ontology–based (GO-based) enrichment analysis performed under the 4 categories Biological Processes, Cellular Components, Molecular Functions, and Gene Sets (Supplemental Figure 4, B–E show the topmost enriched GO terms; Supplemental Data File 3 has the complete list) identified the downregulation of genes related to molecular transmembrane transporter activity, fat-soluble vitamin metabolic process (related to retinoic acid and vitamin D), ion homeostasis (Supplemental Figure 4, B and D), and apical and basal plasma membranes (Supplemental Figure 4, C and E, and Supplemental Figure 5A). The upregulated DEGs were enriched in GO terms related to ECM and cytoskeleton arrangement (Supplemental Figure 4, C and D). Using Reactome pathway analysis (Supplemental Data File 3), the top downregulated proteins included the canonical retinoid cycle in vision and solute carrier–mediated transmembrane transport (Supplemental Figure 5A), while the top upregulated DEGs included kinesins, collagen biosynthesis, and modifying enzymes (Supplemental Figure 5A). Human Phenotype Ontology enrichment analysis (Supplemental Data File 3) identified chorioretinal atrophy, RPE atrophy, and progressive night blindness (Supplemental Figure 5B). Enrichment analysis with LRP2 as a common factor (Supplemental Data File 4) showed enriched GO terms related to the apical plasma membrane, solute transmembrane transporters, fat-soluble vitamin metabolic processes, sensory perception, and steroid metabolic processes (Figure 7D). Upregulated enriched GO terms were related to the cytoskeleton and collagen-containing ECM (Supplemental Data File 4). Reactome pathway analysis identified 3 downregulated pathways: visual phototransduction, metabolism of steroids, and sensory perception (Figure 7E). Table 6 recapitulates selected GO gene sets and their potential link with myopia and PS development.

### Light and cortisol regulate LRP2 expression in human iRPE cells.

**Figure 8:**
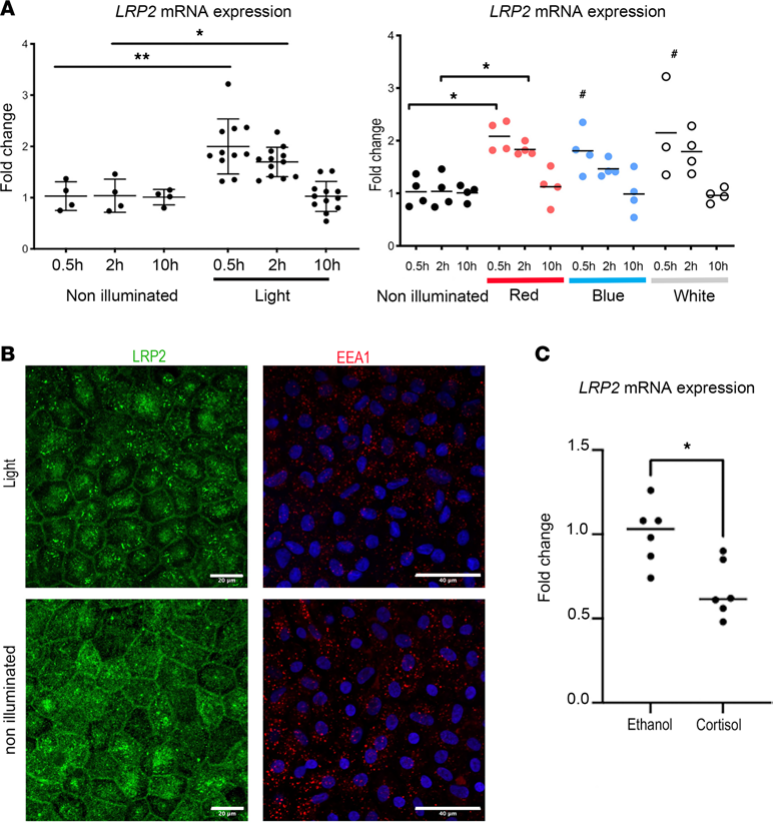
LRP2 expression and environmental factors. (**A**) Quantification of *LRP2* mRNA by qPCR in iRPE not exposed (ne) or exposed to light (left panel) after 0.5, 2, or 10 hours after illumination. Left panel showing quantification of *LRP2* mRNA in iRPE not exposed or exposed to light. Right panel showing quantification of *LRP2* mRNA in iRPE not exposed or exposed to red, blue, or white light. Values correspond to the means of 4 independent experiments in duplicate for each condition. Each independent experiment represents the mean of 3 wells. Data are expressed as fold change in gene expression ± SD and were analyzed using the nonparametric Kruskal-Wallis test and Mann-Whitney post hoc test. *P < 0.05; **P < 0.01; ^#^*P* > 0.05 (not significant). (**B**) LRP2 and EEA1 expression in iRPE not exposed or exposed to red light. (**C**) *LRP2* mRNA expression in iRPE cultures treated without (ethanol) or cortisol. Values correspond to the mean of 6 experiments in triplicate. Data represent the mean fold change in gene expression ± SEM. Mann-Whitney *U* test. *P < 0.05. Scale bars: 20 μm (LRP2) and 40 μm (EAA1).

Light and circadian rhythms are major environmental factors associated with the incidence and the progression of myopia (27) and cortisol secretion, which is under circadian regulation, is modulated by light exposure (28). We assessed the exposure of iRPE cells to an LED system emitting red (631 nm), blue (454 nm), and hot white (3300K) light at a dose of 0.3 J/cm², which was previously shown to be safe for the iRPE (29). After 0.5 and 2 hours of exposure to any of these LEDs, the expression of *LRP2* mRNA increased significantly (fold change 2.00 ± 0.16, *P* = 0.0059 and 1.70 ± 0.08, *P* = 0.0132) and transiently, returning to baseline at 10 hours (fold change 1.02 ± 0.18, *P* = 0.9791) (Figure 8A). Exposure to red light was the most effective at increasing *LRP2* mRNA at 0.5 and 2 hours (fold change 2.08 ± 0.14, *P* = 0.0286 and 1.83 ± 0.06, *P* = 0.0286) (Figure 8A). On immunohistochemistry, 2 hours after illumination, we observed a parallel increase in LRP2 and EEA1 in iRPE cells (Figure 8B).

Having previously demonstrated that the glucocorticoid receptor NR3C1 (GR) and the mineralocorticoid receptor NR3C2 (MR) are both expressed and functional in the iRPE cells used in this study (30), we evaluated the effect of cortisol on *LRP2* expression. Exposure of iRPE cells to 100 nM cortisol led to a significant decrease in *LRP2* mRNA expression after 24 hours (fold change 1.00 ± 0.04 vs. 0.67 ± 0.07; *P* = 0.0152) (Figure 8C).

Interestingly, light also induced the regulation of the *NR3C1* and *NR3C2* genes in iRPE cells. Both white and blue light increased the expression level of *NR3C1* as compared with non-illuminated cells (fold change 1.1 ± 0.1 vs. 1.5 ± 0.4 [*P* = 0.01] and vs. 1.8 ± 0.18 [*P* > 0.0001]), but not red light (Supplemental Figure 6), suggesting that blue light induces RPE cells’ sensitivity to cortisol to a greater extent. *NR3C2* expression was increased similarly by red and blue light (fold change 1 ± 0.2 vs. 2 ± 0.6 and 2 ±1.4; *P* = 0.0001 for both).

Both light exposure and cortisol regulate the expression of *LRP2* in iRPE cells but in an opposite manner, suggesting that LRP2 could be one of the molecular links between environmental factors and HM development.

## Discussion

The findings of this study indicate that reduced levels of soluble LRP2 in the vitreous, and decreased cellular LRP2 in the retina and RPE cells, are associated with NSHM with PS. This suggests that both the membrane-bound and the extracellular form of LRP2 are decreased in myopic eyes with PS. Prior studies have measured LRP2 in the human vitreous proteome (31); however, no association with PS or with myopia has been reported. In a recent study, proteomic analysis of pathologic myopia was performed on 5 HM eyes, but only 2 eyes out of 5 showed PS. However, a subsequent comparison of LRP2 levels between the HM and emmetropic individuals revealed no significant disparities (32), suggesting that the observed decrease in LRP2 levels might be exclusive to PS.

The presence of a soluble form of LRP2 has been detected in renal cells. This soluble form is hypothesized to result from the secretion of an isoform that lacks the cytoplasmic domain. Alternatively, the soluble form may be the result of a 2-step cleavage process involving γ-secretase for intramembrane cleavage and metalloproteases for extracellular cleavage (33). The detection of LRP2 in the vitreous and the plasma membrane/endocytic apparatus of retinal cells in both control and myopic individuals may suggest that extracellular LRP2 cleavage commonly occurs in the ocular tissues. The exact functions of the soluble form of LRP2 still remain to be understood. It could act as a ligand trap, masking or enhancing signaling pathways such as that of SHH (34) or BMP4 that was associated with HM in genetic studies (35).

At the cellular level, LRP2 was predominantly expressed in the apical and basal extremities of RMG cells within the human retina. Recent studies have revealed that LRP2 is also expressed in brain astrocytes and microglia, with its expression being differentially regulated by inflammatory signals and implicated in amyloid β endocytotic activity (36). In the RPE, LRP2 was found to be located at the apical membrane and in vesicles, following a decreasing gradient along the apicobasal axis, ensuring transport between the subretinal space and the choroid, as previously suggested. Additionally, we identified LRP2 signal at the basal side underneath the RPE in the normal human retina, where it could bind to CLU and apolipoproteins and metalloproteases, which are abundant in Bruch’s membrane in humans (37). LRP2 expressed at the basolateral side may also contribute to the regulation of nutrient diffusion and waste disposal between the RPE and blood, as well as to eye homeostasis and growth (18, 38). In NSHM retinas and RPE, a decrease in LRP2 expression was observed in RGM cells and along the endocytic apparatus of the RPE, indicating a potential defect in LRP2-dependent endocytosis. In the proteomic analysis of the vitreous, 9 of the 18 proteins that were increased in myopic eyes have been identified as ligands of LRP2 (see bold font in Tables 2 and 3). Furthermore, CLU and APOE levels were elevated, suggesting that decreased LRP2 expression could modify the traffic of proteins between the vitreous, the retina, the RPE, and the choroid.

A further noteworthy observation is that a significant number of DEPs (7 out of 72 proteins) have been implicated in syndromic and pathological myopias in these vitreous samples with PS, including S-arrestin, versican, lumican, dystroglycan 1, IRBP, and collagen IX α2, which is associated with Stickler syndrome (Supplemental Table 1). However, these proteins are not modified in the vitreous of myopic patients without staphyloma (32). Furthermore, a reduction in neuronal proteins involved in retinal function and synapse function has been observed, suggesting a potential association between PS and retinal pathology. To further investigate the relationship between myopia, SP, and LRP2 expression in RPE cells, we analyzed the transcriptomic changes induced by partial knockdown of LRP2 in iRPE.

The present study demonstrates that even partial downregulation of LRP2 in iRPE induces transcriptional modifications, with genes coding for proteins involved in membrane and vesicular transport being regulated, but also genes coding for proteins involved in the visual cycle (such as RPE65 or retinal dehydrogenase 5 or 11) being downregulated. This suggests that the downregulation of LRP2 could influence the differentiation of RPE cells. The most significantly downregulated biological process in these cells was retinol metabolic process. The association of loss of IRBP function with retinal dystrophy and HM in animal models (39, 40) and humans (41) suggests that retinoic acid could be one of the myopia targets regulated by LRP2. Recent studies have demonstrated that retinoic acid has the capacity to stimulate the production of BMP2 in RPE cells (42). In addition, LRP2 has been identified as an antagonist of BMP signaling (23). Further exploration of the relationship between LRP2, retinoic acid, and BMP is warranted. Furthermore, one study revealed a disruption in the metabolic processes of ions and steroids, with a particular emphasis on the metabolism of vitamin D, that has been associated with myopia in human studies (43). The findings of this study are summarized in Table 6, which outlines the pathways affected by LRP2 downregulation in iRPE and their implications for ECM remodeling, the choroid, the visual cycle, RPE cell polarity and their transport function. It is noteworthy that several pathways regulated in the RPE have the capacity to influence the structure of the sclera and choroid, thus explaining a possible role in the development of PS.

In addition to the differentially apically expressed solute transporters in the RPE, we found numerous genes involved in maintaining the epithelial apical scaffold, such as occludin or myosin VIIA. Both in the *Foxg1-Cre*-*Lrp2^fl/fl^* mouse eye and in human eyes with PS, RPE cell structure and polarity were severely altered, which could be a consequence of ocular elongation, but also an early and possibly LRP2-driven event, as shown in form-deprived myopia in chicks. In the latter, an increase in the surface area of individual RPE cells compensated for the expanded vitreous chamber (44). Similarly, in the *Foxg1-Cre*-*Lrp2^fl/fl^* mouse, we showed in previous studies that the total surface area of the RPE layer was expanded without RPE proliferation or cell death (16, 45). We observed that LRP2 deficiency leads to a severe reduction in clathrin-mediated endocytotic vesicles in humans and in iRPE cells, together with a reduction in EEA1 and LAMP1 vesicles. We could thus speculate that the loss of LRP2 in RPE cells could induce cell surface enlargement through failure of apical membrane removal. As a result of surface expansion, especially posterior to the equator, the RPE would then produce more Bruch’s membrane, which is consistent with morphometric changes in Bruch’s membrane in the development of myopia in humans (46). LRP2 expressed in RMG cells could also contribute to myopia through the regulation of multiple extracellular protein levels through transport mechanisms, including the metabolism of retinoids. Different myopic phenotypes could result from the decreased expression of LRP2 in different cells and timing in the evolution of the disease, as several studies showed that staphyloma develops in HM eyes over time and progresses (3, 47, 48). However, further studies are required to better understand the mechanisms linking LRP2 expression in RPE cells and RMG cells to the development of myopia and PS.

The cause of LRP2 downregulation in myopic eyes is most probably multifactorial and could result from tissue-scale mechanical stresses (49) occurring during excessive eye growth, from genetic or epigenetic predispositions, from a local inflammatory microenvironment (50, 51), from hormonal factors, and from environmental stimuli such as lighting conditions. *LRP2* expression is tightly regulated and its reduction in epithelial cells has been associated with different fibrosis pathologies. Furthermore, this inhibition of transcription is caused by the canonical TGF-β1/SMAD2-SMAD3 pathway (52). Among the factors that positively control *LRP2* are retinoic acid (53), vitamin D (53), and α and γ peroxisome proliferator–activated receptors (54).

Interestingly, our results show that *LRP2* expression is upregulated in iRPE by light exposure, which can be explained by the presence of melanopsin (Opn4) (55, 56) and other non-visual opsins like neuropsin (Opn5) (57) or peropsin (58) in RPE cells. *LRP2* upregulation by light is in line with epidemiological data showing that light exposure is a robust environmental stimulus in the prevention of myopia (59). Our experiments indicated that exposure to red light seemed to upregulate *LRP2* more efficiently than blue or white light, in line with intervention studies in children showing reduced myopia progression by red light therapy (60). An indirect mechanism of action of light could be the circadian regulation of cortisol that is sensitive to the wavelength composition of environmental lighting (28). Interestingly, cortisol downregulated the expression of LRP2 in iRPE cells and blue light, more than red light, appears to be capable of increasing GR expression levels, suggesting that blue light could increase the sensitivity of these cells to cortisol. In a Chinese population, higher levels of cortisone, corticosterone, and aldosterone were recently measured in aqueous humor of myopic eyes (61), suggesting a link between myopia and ocular metabolism of corticoids. In a model of negative lens–induced myopia in guinea pigs, hydrocortisone enhanced the axial elongation, the myopic shift, and scleral thinning but had the reverse effect during physiologic emmetropization (62), demonstrating the potential differential effects of corticoids in emmetropization process and in pathologic myopia. The complex links and the interplay between light, circadian rhythm, corticoids, LRP2, and myopia require further investigations but our results tend to indicate that LRP2 could be a molecular link between light, circadian regulations, and NSHM.

This study is subject to certain limitations, including the relatively small number of human eyes with PS that were available for analysis due to the rarity of such fresh postmortem eyes in biobanks. However, the observation that LRP2 was not only reduced in the retina but also in the vitreous lends further support to our findings. The number of vitreous samples was also limited; however, this study has examined a substantial number of vitreous samples from HM patients with PS, and the findings have been validated by the reproducibility of the results by 2 independent groups in 2 distinct patient populations. Subsequent studies should aim to specifically analyze the relationship between LRP2 in the vitreous, axial length, and PS, and to characterize the soluble LRP2 form.

In conclusion, our study demonstrates that in human eyes with NSHM associated with PS, which is the more severe form of myopia, LRP2 is decreased both in the vitreous and in the RPE. In human RPE cells, LRP2 expression is regulated by light, which is the environmental factor that is most associated with myopia. Finally, the silencing of *LRP2* in human RPE cells regulated the expression of genes involved in myopia development. LRP2 appears as a potential interventional target in the prevention of myopic staphyloma and its blinding complications.

## Methods

### Sex as a biological variable.

Our study included both female and male patients (Table 1). Sex was not considered as a biological variable. Our studies examined male and female mice; similar findings were found in both sexes.

### Ethics — patient recruitment.

A total of 25 patients with NSHM and 23 patients with emmetropic eyes, for which vitrectomy was scheduled for macular surgery or for intraocular lens luxation, were recruited in the study. There were 25 Japanese patients and 23 White European patients. Undiluted vitreous humor samples from eyes with NSHM complicated by PS (*n* = 15) and from emmetropic control eyes (*n* = 10) were obtained for mass spectrometry analysis at Kyoto Prefectural University of Medicine (Table 1). Undiluted vitreous humor samples from eyes with NSHM and PS (*n* = 10) and from emmetropic control eyes (*n* = 10) were collected for ELISA analysis at Hôpital Cochin Ophtalmopole, Paris, France and at Hospital Puerta de Hierro-Majahonda, Albacete, Spain (Table 1). All samples were stored at –80°C until preparation was initiated.

### Sample preparation for mass spectrometry.

Protein concentrations were measured on undiluted vitreous samples with an infrared spectrometer (Direct Detect) and samples were prepared for analysis using the S-Trap Micro spin column digestion protocol from ProtiFi, as described previously in detail (63). Analysis was performed on an Orbitrap Fusion Tribrid mass spectrometer coupled to a Dionex UltiMate 3000 RSLC nano system (Thermo Fisher Scientific). All details are described in the Supplemental Methods.

### ELISA protein quantification of LRP2.

LRP2 concentration was determined by ELISA (Creative Diagnostics, DEIA-FN834). Briefly, microtiter plates were coated with 1 μg polyclonal antibody specific for human LRP2. An undiluted 100 μL of aqueous or vitreous humor sample was added to the plates. The presence of LRP2 was revealed by incubation with the same antibody labeled with biotin. The intensity of this colored product is directly proportional to the concentration of LRP2 present in the samples and was measured immediately by absorbance at 450 nm.

### Eye samples.

Four donor eyes were collected less than 10 hours after death and fixed in 4% paraformaldehyde (PFA) in PBS for 24 hours. Two male donor eyes with moderate cataract and refraction of –0.75 (+0.75) 5° in the right eye and –0.50 (+1) 170° in the left eye were used as emmetropic control eye. The donor, an 88-year-old male with no remarkable medical history and no known ocular diseases, died due to small bowel obstruction. The postmortem time before enucleation and fixation was 6 hours. Both HM eyes from an 89-year-old male donor were analyzed. Medical history reported hypertension and arthritis; the cause of death was pulmonary edema, and postmortem time was 8 hours. Both eyes were pseudophakic and showed macular staphyloma, vitreomacular traction, and degenerative macular schisis. Premortem examination of the left eye was impaired by corneal opacification secondary to corneal surgery for keratoconus. Eyes were fixed in 4% PFA overnight, rinsed, and preserved in 1% PFA at 4°C. Eyes were then dissected to remove the cornea and the anterior segment. The posterior segment was included in optimal cutting temperature compound and 10-μm cryosections were prepared. For flat mounting, after removing the cornea and lens, the remaining posterior segment of the eye was flat mounted and dissected to remove the neural retina. The posterior segment was cut into 4 quarters, and the RPE/choroid was separated from the sclera after resection of the vortex veins.

### Immunostaining of human tissues.

Sclera-choroid RPE complexes were incubated for 1 hour in a solution (0.1 M PBS, 10% normal goat serum, 0.01% Triton X-100) at room temperature. Then, tissues were incubated with the primary antibodies at appropriate dilution (Supplemental Table 3) and phalloidin-rhodamine (1:200, Thermo Fisher Scientific) in a buffer solution (0.1 M PBS, 5% normal donkey serum, 0.01% Triton X-100) for 5 days at 4°C under gentle agitation. After six 30-minute consecutive washes, tissues were incubated for 3 hours at room temperature with the appropriate secondary antibodies diluted 1:1000. After four 30-minute successive washings, tissues were flat mounted using Dako Omnis Fluorescence Mounting Medium (Agilent). A similar protocol was applied for immunostaining on transversal cryosections, with overnight (4°C) incubation for the primary antibody and 1 hour (at room temperature) for secondary antibody incubation. Images were acquired using a fluorescence microscope (Olympus BX51) or a confocal microscope (Zeiss LSM 710).

### iRPE cell culture and differentiation.

An hiPSC line, obtained from fibroblasts of a healthy donor, was used as previously described (30). The hiPSCs were expanded and differentiated into iRPE cells using a differentiation protocol (30). All details of cell culture and maintenance are described in the Supplemental Methods.

At passage 3, iRPE cells were seeded in Transwells (0.4 μm pore polyester membrane inserts, Corning, CLS3450 and CLS3460) coated with Matrigel phenol red–free matrix (Corning, 356231) in a serum- and antibiotic-free Retinal Differentiation Medium (Supplemental Methods). All cultures were maintained in an incubator at 37°C and 5% CO_2_. The transepithelial resistance (TER) was measured (Supplemental Methods), and cells were used between the sixth and the tenth week of culture, once the TER reached physiological levels (>100 Ω/cm^2^).

### RNA silencing.

Briefly, for each transfection, 6 μL of triplex siRNA, a pool of 3 target-specific 19- to 25-nucleotide-long *siLRP2* (Santa Cruz, sc-40103) or scrambled siRNA (Santa Cruz Biotechnology, sc-37007) or fluorescein-conjugated control siRNA (Santa Cruz Biotechnology, sc-36869) was diluted in 100 μL of siRNA transfection medium (Santa Cruz Biotechnology, sc-36868). This solution was mixed v/v with a diluted solution of siRNA transfection reagent (Santa Cruz Biotechnology, sc-29528). The siRNA mixture was added to the apical surface of iRPEs growing on Transwells for 6 hours. Without removing the siRNA mixture, serum-concentrated medium (2×) was added for an 18-hour incubation. After incubation, the cells were placed in fresh medium for 24 hours. The cells were then collected for protein and RNA extraction.

### RNA sequencing.

Raw data quality was assessed using FastQC (https://www.bioinformatics.babraham.ac.uk/projects/fastqc/). Low-quality sequences and adapters were pruned or removed using Illumina’s DRAGEN Bio-IT platform (v3.10.4) with default settings to retain high-quality matched reads. DRAGEN was used for mapping to the hg38 reference genome and quantification using the Gencode v37 annotation GTF file. Library orientation, composition, and transcript coverage were checked using Picard tools (https://broadinstitute.github.io/picard/). Subsequent analyses were performed using R software (https://www.r-project.org/). Data normalization was performed using DESeq2 (v1.26.0) Bioconductor software package (DESeq2: https://bioconductor.org/packages/release/bioc/html/DESeq2.html), followed by differential expression analysis using the DESeq2 workflow. Adjusted *P* values for multiple hypotheses were calculated using the Benjamini-Hochberg procedure to control the false discovery rate (FDR). Finally, an enrichment analysis was performed using the R clusterProfiler (v3.14.3) package for Gene Set Enrichment Analysis (GSEA) on gene sets from the C5 ontology, comprising GO grouping biological processes, cellular components, and molecular functions, and Human Phenotype Ontology (http://geneontology.org/; last access on April 18, 2024), and the KEGG (https://www.genome.jp/kegg/) and REACTOME (https://reactome.org/) databases. The raw and processes data used for this study have been deposited in the NCBI Gene Expression Omnibus (GEO) with accession number GSE277911.

### Light exposure.

Cells were exposed to red (630 nm, Qasim1xQA-SL0001-EU, Qasim LED), blue (454 nm, Joyland, D50SWD-B), or white (3300K, Lepro, 4100067-WW-EU-NF-a) LEDs in a 37°C incubator with 5% CO_2_. The specificities of each light source (correlated color temperature, peak of emission, integrated irradiance) were measured using a spectroradiometer (Konica Minolta CL70 F CR I). Cells were exposed to a white, blue, and red LED for 820, 1000, and 900 seconds, respectively, to match ambient light exposure. These exposure times correspond to a dose of 0.3 J/cm^2^ (calculated as the energy of the integrated spectrum multiplied by the exposure time), as previously shown (64) (light spectra are provided in Supplemental Figure 7). Cells were illuminated for 5 days, and mRNA collected at 3 postillumination times: 30 minutes, 2 hours, and 10 hours. TER measurements were taken during the blue-light illumination protocol to assess cell integrity during the experiment.

### Corticosteroid treatment of iRPE cells.

Cells were seeded at passage 3 in cell culture plastic dishes. On day 35, one week prior to corticosteroid treatment, retinal differentiation medium was removed and iRPE cells were incubated in experimental corticosteroid-free medium (DMEM, high glucose, HEPES, no phenol red [Thermo Fisher Scientific]; 10% fetal bovine serum, charcoal stripped [Thermo Fisher Scientific]). On day 42, iRPE cells were treated for 24 hours with cortisol (1 × 10^–7^ M). As cortisol was dissolved in ethanol, control cells were treated with 0.1% ethanol in medium.

### RT-qPCR.

Total RNA was isolated using commercially available kits according to the manufacturer’s instructions (RNeasy Mini, Qiagen) and measured (Nanodrop, Peqlab). One milligram was used in a reverse transcription reaction (SuperScript First strand synthesis, Thermo Fisher Scientific). qPCR was performed using Master Mix PCR Power SYBR Green (4367659, Applied Biosystems) on a 7500 real-time PCR system (Applied Biosystems). Transcript levels were calculated using the standard curves generated using serial dilutions of cDNA obtained from samples, which were then normalized to *HPRT*. Primer sequences are listed in Table 6. Each plotted value corresponds to the mean of 3 independent experiments in duplicate.

### Immunocytochemistry.

iRPE cells were fixed with 4% PFA. Primary antibodies were incubated in blocking buffer (1% BSA, 0.1% Triton X-100 in PBS) for 3 hours. Secondary antibodies were incubated for 1 hour. Nuclei were counterstained with DAPI for 5 minutes. Antibodies and reagents are listed in Supplemental Table 3. No cellular autofluorescence or nonspecific labeling was detected under these conditions. Images were collected by confocal microscopy and processed using ZEN (Zeiss) and ImageJ (NIH) software.

Eyes were enucleated (*n* = 7 WT, *n* = 7 *Lrp2*-cKO; age = 20 weeks) and fixed for 30 minutes in 4% PFA. The RPE was dissected and laid flat, then postfixed with acetone for 10 minutes at –20°C. Tissues were incubated with phalloidin for 3 hours, rinsed, and then incubated with rabbit anti–ZO-1 in blocking buffer (1% BSA, 0.3% Triton X-100 in PBS) for 24 hours at 4°C. Secondary antibody was incubated 2 hours at room temperature. After mounting, RPE was cut at 4 spots, yielding 4 quadrants corresponding to the nasotemporal and dorsoventral directions. Images were acquired using an inverted confocal microscope (Zeiss LSM 710). Flat mounts were imaged with a 20× objective lens. Typically, 3 to 4 images were obtained of each of the 4 quadrants to cover studied regions (intermediate or peripheral retina). Using ImageJ software, a macro was developed to calculate the RPE cell areas for each sample. The macro is divided into 3 main stages: the first stage involves segmentation, including image binarization and skeletonization; the second stage involves manual ROI correction; and the final stage involves skeleton analysis, requiring the following plugins: tubeness, morphology, and analyze skeleton. To calculate the RPE cell density, the total area of RPE cells per region of interest (intermediate or periphery) was divided by the total number of RPE cells per region of interest.

### Statistics.

The data represent the mean ± SD. The nonparametric Kruskal-Wallis test followed by the Dunn’s post hoc test that corrects for multiple comparisons was used when more than 2 groups were compared and the Mann-Whitney *U* test was used to compare 2 groups with Prism 8 software (GraphPad Software). A *P* value of less than 0.05 was considered significant.

### Data availability.

All data analyzed in this study are included in the published article. Values for all graphs and supplemental graphs are reported in the Supporting Data Values file. The raw and processed data used for RNA sequencing analysis have been deposited in the NCBI GEO with accession number GSE277911.

### Study approval.

The proteomic study on vitreous samples was conducted in accordance with the Institutional Review Board of Kyoto Prefectural University of Medicine (permission RBMR-C-864-6), and all patients provided written informed consent. The ELISA LRP2 test was performed on vitreous samples in France and Spain, where vitreous samples are classified as surgical waste and can be utilized for research purposes if patients have not expressed any opposition, as per French and Spanish law. All patients provided written consent for the use of surgical waste for research purposes. Storage and analysis were authorized in France by CPP Ile de France 1 (N°2016-14390), and the use of patients’ information received authorization number CNIL 2233436.

Four donor eyes were obtained by Lion’s Gift of Sight, which operates under the rules of the Food and Drug Administration and the Eye Bank Association of America. Donor consent was obtained, and the next of kin received no financial compensation or benefit from the donation. The importation of the tissue into France was carried out in accordance with the regulations that apply to the transfer of human tissues. This process was authorized by the French Ministry of Research with authorization number IE-2022-2285.

## Author contributions

KD, AT, OC, and FBC conceived and designed the study and wrote the original draft of the manuscript. KD, E Picard, PL, LJ, VC, and E Pussard performed experiments. RK provided *Lrp2*-cKo mice. JMRM and JRM provided vitreous samples. KK collected vitreous samples for mass spectrometry and contributed to the analysis of proteomics data. HV, BH, and LJC performed mass spectrometry and data analysis of the proteomics data. All authors contributed to reviewing and editing of the manuscript and approved the submitted version.

## Supplementary Material

Supplemental data

Unedited blot and gel images

Supporting data values

## Figures and Tables

**Table 2:**
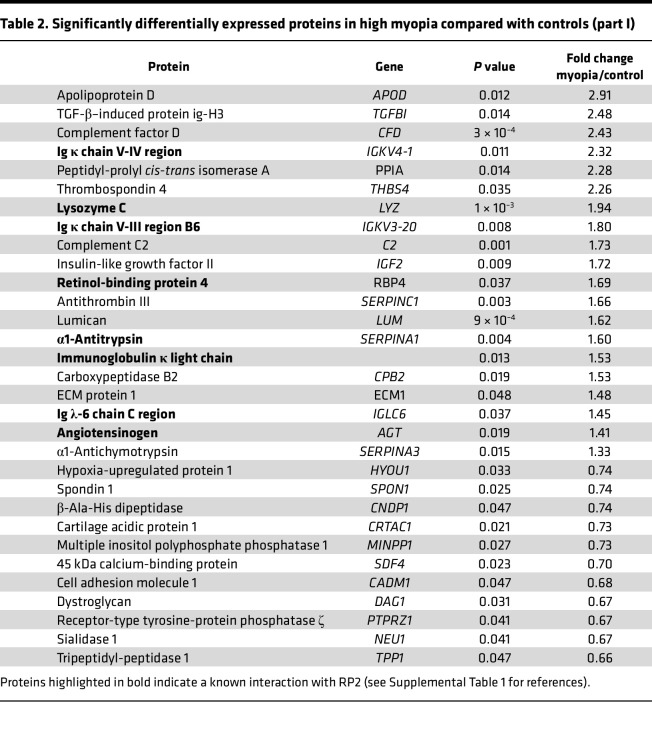
Significantly differentially expressed proteins in high myopia compared with controls (part I)

**Table 1:**
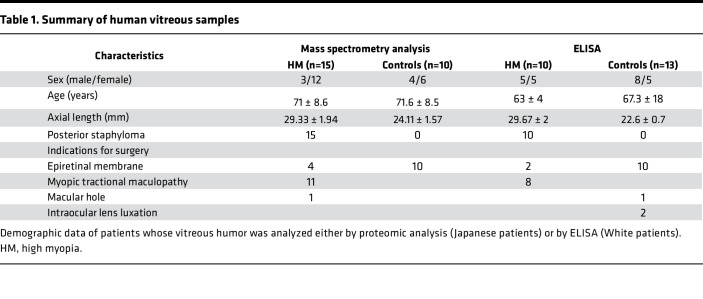
Summary of human vitreous samples

**Table 3:**
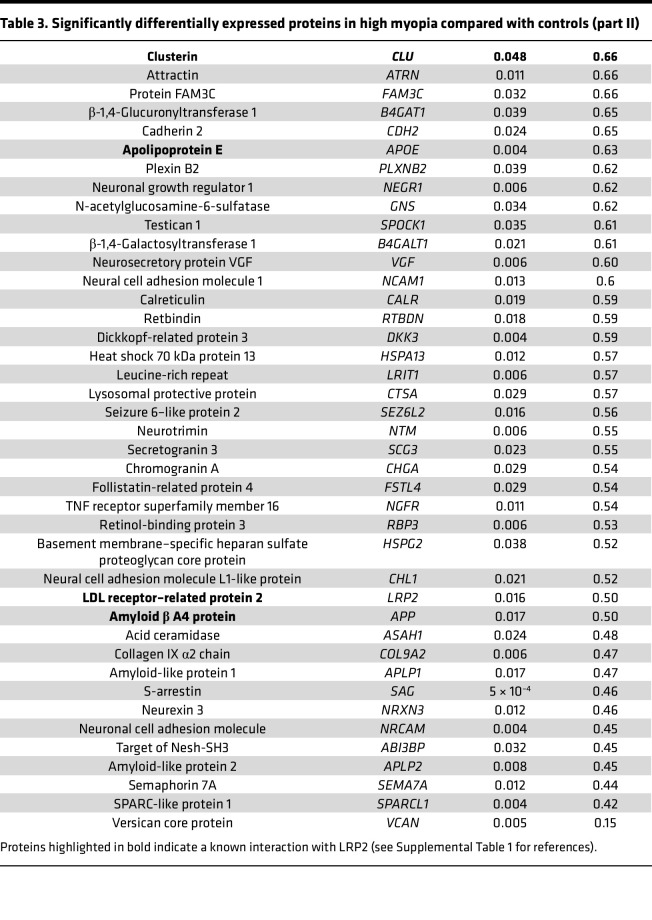
Significantly differentially expressed proteins in high myopia compared with controls (part II)

**Table 4:**
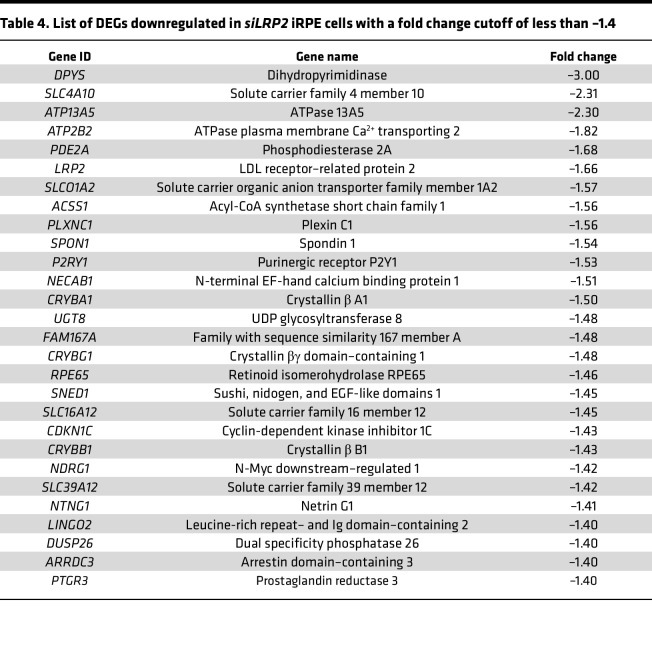
List of DEGs downregulated in *siLRP2* iRPE cells with a fold change cutoff of less than –1.4

**Table 5:**
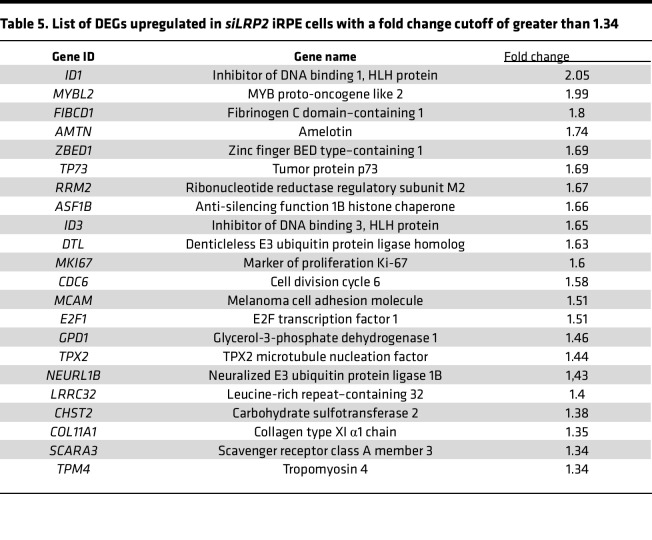
List of DEGs upregulated in *siLRP2* iRPE cells with a fold change cutoff of greater than 1.34

**Table 6:**
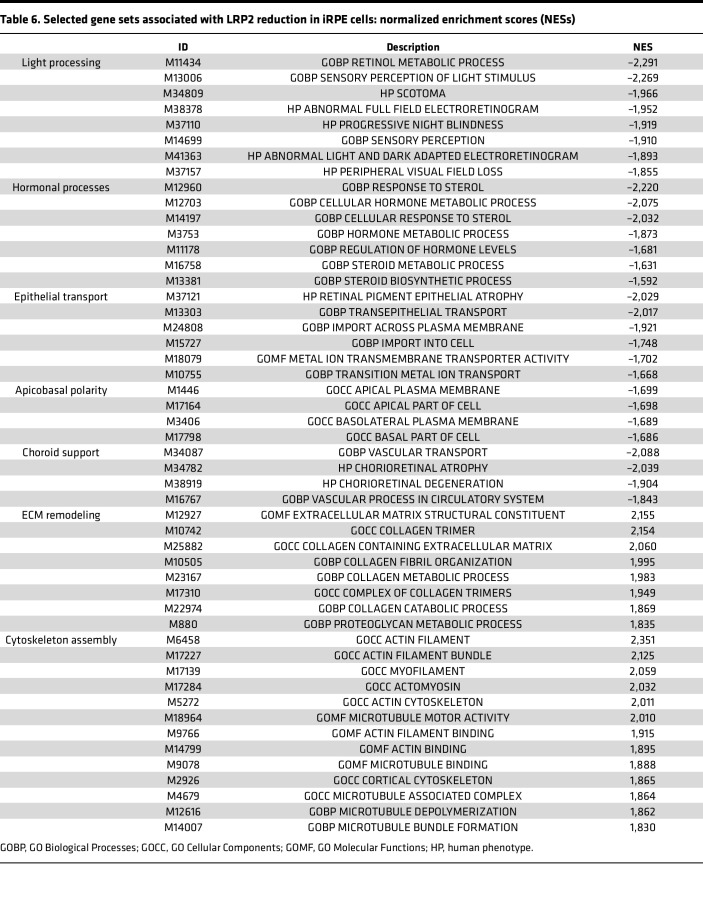
Selected gene sets associated with LRP2 reduction in iRPE cells: normalized enrichment scores (NESs)

## Version 1 06/24/2025

In-Press Preview

## Version 2 08/08/2025

Electronic publication

## Version 3 08/13/2025

Author name correction. The 10th author is Jorge Ruiz-Medrano.
