# Supplementary material for: LRP2 is a potential molecular target for nonsyndromic pathological myopia
Source: JCI Insight. 2025 Jun 24;10(15):e192929. doi: 10.1172/jci.insight.192929 (PMC12333944; doi:10.1172/jci.insight.192929)
Supplement: Supplemental data [file jciinsight-10-192929-s015.pdf]

Supplementary Materials

LRP2 is a potential molecular target for pathological myopia

Kimberley Delaunay[1], Emilie Picard[1], Patricia Lassiaz[1], Laurent Jonet[1], Vidjea Cannaya[1], José Maria Ruiz Moreno[2], Kentaro Kojima[3], Henrick Vorum[4,5], Bent Honoré[5,6], Jorge R Medrano[2], Lasse Jorgensen Cehofski[4,5], Eric Pussard[7], Renata Kozyraki#[8], Alicia Torriglia #[1], Olivier Cases [1#,] Francine Behar-Cohen#[1,9].

Supplementary Tables

Supplementary Table 1: List of differentially expressed genes (DEGs) down regulated in *shLRP2* iRPE cells with a fold change cut off of less that 1.1.

Supplementary Table 2: List of DEGs up regulated in *shLRP2* iRPE cells with a fold change greater than 1.1.

Supplementary Table 3: Antibodies and reagents for immunohistochemical staining.

Supplementary Material and methods

5 Supplementary Figures

Supplementary Figure 1: GO analysis of DEPs

Supplementary Figure 2: The most significant cluster obtained from the PPI and its disease enrichment

Supplementary Figure 3: Principal component analysis

Supplementary Figure 4: KEGG, and Gene Ontology based gene enrichments

Supplementary Figure 5: Reactome and Human Phenotype Ontology based gene enrichments

Supplementary Figure 6: NR3C1 and NR3C2 mRNA expression upon lighting conditions

Supplementary Figure 7: Emission spectra of the LED lights used in the study.

Supplemental Table 1: List of differentially expressed genes (DEGs) down regulated in *shLRP2* iRPE cells with a fold change cut off of - 1.1>.

Gene ID	Gene Name	Fold	Additional informations
DPYS	dihydropyrimidinase	-3.00	Second step of reductive pyrimidine degradation - Dihydropyrimidinase deficiency (DPYSD) associated with congenital microvillous atrophy
SLC4A10	solute carrier family 4 member 10	-2.31	Sodium-driven chloride bicarbonate exchanger – NEDHBA syndrome
ATP13A5	ATPase 13A5	-2.30	ATPase
ATP2B2	ATPase plasma membrane Ca2+ transporting 2	-1.82	ATP-driven Ca2+ ion pump
PDE2A	phosphodiesterase 2A	-1.68	cGMP-activated cyclic nucleotide phosphodiesterase – IDDPADS syndrome
LRP2	LDL receptor related protein 2	-1.66	Multiligand endocytosis receptor – Apical membrane RPE
SLCO1A2	solute carrier organic anion transporter family member 1A2	-1.57	Na+-independent transporter – Mediates apical uptake of all-trans-retinol across human RPE
ACSS1	acyl-CoA synthetase short chain family 1	-1.56	Catalyzes the synthesis of acetyl-CoA from short-chain fatty acids
PLXNC1	plexin C1	-1.56	Receptor for SEMA7A
SPON1	spondin 1	-1.54	Cell Adhesion Molecule
P2RY1	purinergic receptor P2Y1	-1.53	Receptor for extracellular adenine nucleotides – Degradation of ATP by RPE
NECAB1	N-terminal EF-hand calcium binding protein 1	- 1.51	Calcium binding protein
CRYBA1	crystallin beta A1	-1.50	Heat shock protein – Apical membrane RPE, endocytosis
UGT8	UDP glycosyltransferase 8	-1.48	Catalyzes the transfer of galactose to ceramide
FAM167A	family with sequence similarity 167 member A	-1.48	N.D.
CRYBG1	Beta-gamma crystallin domain-containing 1	-1.48	Cytoskeleton
RPE65	retinoid isomerohydrolase RPE65	-1.46	Isomerohydrolase in the retinoid cycle involved in regeneration of 11-cis-retinal – Leber Congenital Amaurosis 2 (LCA2)
SNED1	sushi, nidogen and EGF like domains 1	-1.45	Extracellular matrix molecule
SLC16A12	solute carrier family 16 member 12	-1.45	Creatine transporter – Cataract 47 (CTRCT47)
CDKN1C	cyclin dependent kinase inhibitor 1C	-1.43	Potent tight-binding inhibitor of several G1 cyclin/CDK complexes
CRYBB1	crystallin beta B1	-1.43	Interaction CRYBA1 - Cataract 17, multiple types (CTRCT17)
NDRG1	N-myc downstream regulated 1	-1.42	Stress-responsive protein – Charcot-Marie-Tooth Diseases 4D
SLC39A12	solute carrier family 39 member 12	-1.42	Uniporter that promotes Zn2+ import from the extracellular space to the cytoplasm. Identified as causing non-syndromic high myopia
NTNG1	netrin G1	-1.41	Guidance Molecule
LINGO2	leucine rich repeat and Ig domain containing 2	-1.40	N.D.
DUSP26	dual specificity phosphatase 26	-1.40	Inactivation of MAPK1 and MAPK3
ARRDC3	arrestin domain containing 3	-1.40	Endosome adaptator
PTGR3	prostaglandin reductase 3	-1.40	Functions as15-oxo-prostaglandin 13-reductase
TMEM255A	transmembrane protein 255A	-1.39	N.D.
SLC16A4	solute carrier family 16 member 4	-1.39	Proton-linked monocarboxylate plasma membrane transporter
SELENOP	selenoprotein P	-1.39	Antioxidant defense properties of selenium
LRP8	LDL receptor related protein 8	-1.38	Cell surface receptor for Reelin – Myocardial Infarction 1 (MCI1)
MAN2A1	mannosidase alpha class 2A member 1	-1.38	Biosynthesis of N-glycans
FLVCR2	FLVCR heme transporter 2	-1.37	Heme importer/sensor – Proliferative Vasculopathy and Hydranencephaly-Hydrocephaly syndrome (PVHH) Vasculopathy of the retina
RDH5	retinol dehydrogenase 5	-1.37	11-cis retinol oxidation in the RPE – Fundus Abipunctatus (FALBI) – Flecked retina, night blindness
CYP4V2	cytochrome P450 family 4 subfamily V member 2	-1.36	Cytochrome P450 monooxygenase involved in fatty acif metabolism – Bietti Crystalline corneoretinal distrophy (BCD) – Degeneration of the retina and sclerosis of choroidal vessels
SOSTDC1	sclerostin domain containing 1	-1.36	Directly antagonizes activity of BMP2, BMP4, and BMP7 – RPE expression
MEST	mesoderm specific transcript	-1.36	N.D.
NOG	noggin	-1.36	Inhibitor of BMP – decreased in the guinea-pig myopic model
BCO1	beta-carotene oxygenase 1	-1.35	Cleavage of beta-carotene in 2 retinal – Hypercarotenemia and Vitamine A Deficiency (HCVAD) increased beta-carotene and decreased vitamine A levels
HSD17B11	hydroxysteroid 17-beta dehydrogenase 11	-1.34	Androgen metabolism during steroidogenesis
RHOU	ras homolog family member U	-1.32	Control of cell shape
S1PR1	sphingosine-1-phosphate receptor 1	-1.30	G-protein coupled receptor for the bioactive lysosphingolipid shingosine-1-phosphate
HNMT	histamine N-methyltransferase	-1.29	Inactivation of histamine – Intellectual Developmental Disorder (MRT51)
RDH11	retinol dehydrogenase 11	-1.29	Retinol deshydrogenase with higher activity towards 9-cis, 11-cis and all-trans-retinol – Retinal Distrophy Juvenile Cataracts, and Short Stature syndrome (RDJCSS) – Retinal distrophy, RPE and choroid atrophy.
DNAJC3	DnaJ heat shock protein family (Hsp40) member C3	-1.28	Endoplasmic reticulum stress – Ataxia, combined Cerebellar and Peripheral, with Hearing loss and Diabetes mellitus (ACPHD)

LEPROTL1	leptin receptor overlapping transcript like 1	-1.28	N.D.
CTNNAL1	catenin alpha like 1	-1.27	Modulation of the Rho pathway
MAB21L2	mab-21 like 2	-1.26	Embryonic development including eye – Microphtalmia/Coloboma and Skeletal Dysplasia syndrome (MCSKS) -
ANKRD46	ankyrin repeat domain 46	-1.25	N.D.
FAXDC2	fatty acid hydroxylase domain containing 2	-1.23	Megakariocyte differentiation
LETMD1	LETM1 domain containing 1	-1.22	Mitochondrial structure
TOB1	transducer of ERBB2, 1	-1.21	Anti-proliferative protein
LIPG	lipase G, endothelial type	-1.21	Hydrolysis of HDL
ST3GAL5	ST3 beta-galactoside alpha-2,3-sialyltransferase 5	-1.21	Formation of gangliosides – Salt and Pepper Developmental Regression syndrome (SPDRS)
STMP1	short transmembrane mitochondrial protein 1	-1.20	Mitochondrial microprotein
EIF4A2	eukaryotic translation initiation factor 4A2	-1.19	Translation initiation factor
SARAF	store-operated calcium entry associated regulatory factor	-1.18	Negative regulator of store-operated calcium entry
FAM13A	family with sequence similarity 13 member A	-1.18	N.D.
SLC20A1	solute carrier family 20 member 1	-1.17	Sodium-phosphate symporter
RHOB	ras homolog family member B	-1.14	Apoptosis
CPE	carboxypeptidase E	-1.14	Sorting receptor that directs prohormones to the secretory pathway – BDV syndrome
BMF	Bcl2 modifying factor	-1.13	Apoptosis

Supplemental Table 2: List of DEGs up regulated in *shLRP2* iRPE cells with a fold change cut off of 1.1<.

Gene ID	Gene Name	Fold	Additional informations
ID1	inhibitor of DNA binding 1, HLH protein	2.05	Transcriptional regulator - Regulates the circadian clock by repressing the transcriptional activator activity of the CLOCK-BMAL1 heterodimer
MYBL2	MYB proto-oncogene like 2	1.99	DNA binding E2F motifs
FIBCD1	fibrinogen C domain containing 1	1.8	Acetyl group-binding receptor facilitating endocytosis
AMTN	Amelotin	1.74	Promoter of calcium phosphate mineralization – Amelogenesis imperfecta 3B (AI3B)
ZBED1	zinc finger BED-type containing 1	1.69	E3-type small ubiquitin-like modifier (SUMO) ligase
TP73	tumor protein p73	1.69	Apoptotic response to DNA damage – Ciliary dyskinesia, primary, 47, and lissencephaly (CILD47)
RRM2	ribonucleotide reductase regulatory subunit M2	1.67	Biosynthesis of deoxyribonucleotides from the corresponding ribonucleotides
ASF1B	anti-silencing function 1B histone chaperone	1.66	Histone chaperone
ID3	inhibitor of DNA binding 3, HLH protein	1.65	Transcriptional regulator - Regulates the circadian clock by repressing the transcriptional activator activity of the CLOCK-BMAL1 heterodimer. Differentially expressed in RPE in a guinea pig model of myopia
DTL	denticleless E3 ubiquitin protein ligase homolog	1.63	Substrate-specific adapter of a DCX (DDB1-CUL4-X-box) E3 ubiquitin-protein ligase complex - regulates the circadian clock function by mediating the ubiquitination and degradation of CRY1
MKI67	marker of proliferation Ki-67	1.6	Binds DNA, with a preference for supercoiled DNA and AT-rich DNA
CDC6	cell division cycle 6	1.58	Initiation of DNA replication – Meier Gorlin syndrome 5 (MGORS5)
MCAM	melanoma cell adhesion molecule	1.51	Cell adhesion molecule
E2F1	E2F transcription factor 1	1.51	Transcription factor
GPD1	glycerol-3-phosphate dehydrogenase 1	1.46	Glycerol-3-phosphate dehydrogenase activity – Hypertriglyceridemia, transient infantile (HTGTI)
TPX2	TPX2 microtubule nucleation factor	1.44	Microtubule nucleator
NEURL1B	neuralized E3 ubiquitin protein ligase 1B	1,43	E3 ubiquitin-protein ligase involved in regulation of the Notch pathway
LRRC32	leucine rich repeat containing 32	1.4	Key regulator of TGF beta signaling
CHST2	carbohydrate sulfotransferase 2	1.38	Inflammation
COL11A1	collagen type XI alpha 1 chain	1.35	Fibrillogenesis. Sticler syndrome 2 (STL2)– Marshall syndrome (MRSHS) – ocular abnormalities consisting of large eyes with ocular hypertelorism- high myopia
SCARA3	scavenger receptor class A member 3	1.34	Protection against oxidative molecules
TPM4	tropomyosin 4	1.34	Binds to actin filaments – Bleeding disorder (BDPLT25)
BSN	bassoon presynaptic cytomatrix protein	1.33	Scaffolf protein in the presynaptic compartment
GRIK3	glutamate ionotropic receptor kainate type 3	1.32	Ionotropic glutamate receptor
NFASC	neurofascin	1.31	Cell adhesion molecule – Neurdevelopmental disorder (NEDCPMD)
ADAM28	ADAM metallopeptidase domain 28	1.28	Adhesion and protolytic events in lymphocyte
PCSK7	proprotein convertase subtilisin/kexin type 7	1.27	Serine endoprotase
FHOD3	formin homology 2 domain containing 3	1.26	Actin-organizing protein
ADAMTS7	ADAM metallopeptidase with thrombospondin type 1 motif 7	1.24	Metalloprotease
TCOF1	treacle ribosome biogenesis factor 1	1.24	Nucleolar protein – Treacher Collins syndrome (TCS1)
PALM	paralemmin	1.21	Plasma membrane dynamics
LEFTY2	left-right determination factor 2	1.21	Left-right asymmetry determination of organs – Left-right axis malformations (LRAM)
MMP14	matrix metallopeptidase 14	1.21	Endopeptidase of the ECM produced by RPE – Winchester syndrome (WNCHS)
ADAMTS12	ADAM metallopeptidase with thrombospondin type 1 motif 12	1.18	Metalloprotease
NDST1	N-deacetylase and N-sulfotransferase 1	1,15	Heparan sulfate biogenesis – Intellectual Developmental disorder (MRT46)
HNRNPL	heterogeneous nuclear ribonucleoprotein L	1,15	Splicing factor

Supplementary Table 3: Antibodies and reagents for immunohistochemical staining.

Primary Antibodies	Species, Label, Method	Source, number	Dilution
Glutamine synthetase	Mouse, IF	Merck Millipore, MAB2-302	1:300
Tubulin-beta3	Mouse, IF	BioLegend, 801202	1:300
OPN1LW/OPN1MW (Red/green-sensitive opsin)	Rabbit, IF	Merck Millipore, AB5405	1:100
OPN1SW (Blue-sensitive opsin)	Goat, IF	Santa Cruz, sc-14363	1:200
Glial fibrillary acidic protein	Rabbit, IF	Dako Cytomation, Z0334	1:300
LRP2	Rabbit, IF	Abcam, Ab76969	1:300
ZO-1	Rabbit, IF	Invitrogen, 40-2200	1:200
Clathrin	Mouse, IF	Santa Cruz, sc-12735	1:200
EEA1	Mouse, IF	BD Transduction, 610457	1:250
LAMP1	Mouse, IF	DSHB, H4A3	1:100
**Secondary Antibodies**			
Anti-Rabbit	Donkey, alexa-488, IF	ThermoFisher, A21206	1:300
Anti-Rabbit	Donkey, alexa-594, IF	ThermoFisher, A21207	1:300
Anti-Mouse	Donkey, alexa-594, IF	ThermoFisher, A32766	1:300
Anti-Mouse	Goat, alexa-594, IF	ThermoFisher, A11005	1:300
**Reagents**			
Phalloidin (Aminata Phalloidin)	Rhodamine, IF	ThermoFisher, R415	1:300
4',6-diamidino-2-phenylindole (DAPI)	Fluorescent counterstain	Roche, 10236276001	1:2500
Vectashield Hardset	Antifade mounting medium	Vector Laboratories	
**Primers**			
Sense 5’-GCCGATGCATTTATCAAAAC-3’	Human, LRP2, QPCR	Eurofins	
Antisense 5’-TCACATCCATCTATCTCC-3’	Human, LRP2, QPCR	Eurofins	
Sense 5’-CATTGCTGAGGATTTGGAAAGG-3’	Human, HPRT, QPCR	Eurofins	
Antisense 5’-CTTGAGCACACAGGGCTACA-3’	Human, HPRT, QPCR	Eurofins	

Supplementary MATERIAL AND METHODS

Quantitative Mass Spectrometry by Label-Free Quantification Nano Liquid Chromatography – Tandem Mass Spectrometry (LFQ nLC-MS/MS). Samples were resuspended in 0.1% formic acid and analyzed with LFQ nLC-MS/MS. Peptides were dissolved to 1.0 µg per µl. One µg of each sample was injected and analyzed in duplicates. Mass spectrometry was performed on an Orbitrap Fusion Tribrid mass spectrometer (Thermo Fisher Scientific Instruments, Waltham, MA, USA) coupled to a Dionex UltiMate 3000 RSLC nano system (Thermo Fisher Scientific Instruments) as previously described (1). The mass spectrometer was equipped with an EasySpray ion source (Thermo Fisher Scientific Instruments). Raw files were searched against the Uniprot *Homo Sapiens* database (www.uniprot.org) using the MaxQuant software version 1.6.6.0 (Max Planck Institute of Biochemistry, Martinsried, Germany; https://maxquant.net/maxquant/).

Mass spectrometry data were filtered in Perseus software version 1.6.2.3 (Max Planck Institute of Biochemistry, Martinsried, Germany; https://maxquant.net/perseus/) as previously described (2, 3). LFQ values were $\log_2$ transformed followed by the calculation of mean LFQ values. Successful identification and quantification in at least 70% of the samples in each group was required.

Statistics. Statistical analysis was performed on proteins with successful identification and quantification in at least 70% of the samples in the HM group and 70% of the samples in the control group. Imputation of missing values was not performed. Statistical analysis by the Student's *t*-test was conducted in Perseus to compare HM with controls.

Network biology and systems level analysis. Proteins identified were entered in STRING database (https://string-db.org/) to create a protein–protein association network. The minimum required interaction score was set to a high confidence level of 0.7. Predicted interactome was evaluated using String software based on annotation enrichment strategy. The process to attach biological information relied on a series of enrichment-based tools including Gene Ontology Resource (GO; geneontology.org), PANTHER16.0 (pantherdb.org), or KEGG.

iRPE cell culture and differentiation. Subconfluent hiPSC cultured on Matrigel® hESC-Qualified Matrix (Corning, France) with mTeSR™1 medium (Stemcell Technologies, Germany) were detached with Collagenase, Type IV (Thermo Fisher Scientific). hiPSC colonies within 50-300 µm diameter range were transferred and cultured into flasks of 25 cm² with Embryoid Body Medium (DMEM/F-12, HEPES medium; 1% N-2; 1% B-27™; 1% L-Glutamine; 0.1 mM 2-Mercaptoethanol; 5 µM RHO/ROCK pathway inhibitor Y-27632 (Stemcell Technologies)), to form three-dimensional embryoid body-like (EB) aggregates. On day 1, EB were plated on cell culture dishes (P60) coated with Matrigel® Matrix Basement Membrane Growth Factor Reduced (Corning) and cultured for 10 days in neural induction medium (NIM) (DMEM/F-12, HEPES medium; 1% N-2; 1% L-Glutamine; 1% MEM Non-Essential Amino Acids Solution; Heparin (Merck Millipore)). On day 10, NIM was replaced and Ebs were cultured during three days in Retinal Differentiation Medium (RDM) (DMEM-high glucose-GlutaMAX™; HEPES medium /Ham's F-12 Nutrient Mix medium [3:1]; 2% B-27™ with vitamin A (all products from Thermo Fisher Scientific). On day 13, Ebs were cultured in RDM. When satisfactory level of pigmentation was reached (by day 30-40), iRPE pigmented foci were manually microdissected under a microscope using a surgical blade, collected, seeded on Matrigel® Matrix Basement Membranec

and grown for an additional 30 days in RDM until deeply pigmented monolayers re-formed. On day 60-70, mature iRPE cells were resuspended in RDM containing 5 μM of RHO/ROCK pathway inhibitor Y-27632 and then reseeded on Matrigel® Matrix Basement Growth Factor Reduced coated 24-well plates for further expansion and maturation. At this stage, iRPE cells were at passage 1 (P1) and cultured in the serum- and antibiotic-free RDM.

Trans-Epithelial Resistance. The trans-epithelial resistance (in Ω/cm^2) was measured using the EVOM2 Epithelial Voltohmmeter and an STX2 electrode (World Precision Instruments, Sarasota, Florida). Each value corresponds to the average of several independent wells and was corrected for background resistance using a blank well with culture medium.

Quantitative RT-qPCR. The *LRP2* target gene was analyzed (Supplementary Table 3). Expression levels of individual gene were normalized with *hprt1* sense *5'-CATTGCTGAGGATTTGGAAAGG -3',* antisense *5'-CTTGAGCACACAGGGCTACA-3'* in the same sample by calculation of the ΔCt value, and relative quantification was performed using the ΔΔCt method with unilluminated iRPEs serving as controls.

Western Blot. Cells from 6 wells (N=6) of iRPE were lysed in RIPA extraction buffer (50 mM Tris HCl pH 8 ; 150 mM NaCl; 1% NP-40; 0.5% sodium deoxycholate; 0.1% sodium dodecyl sulfate) (Thermo Fisher Scientific) for the extraction of membrane, nuclear, and cytoplasmic proteins in the presence of protease inhibitors. Protein concentration was measured using the bicinchoninic acid (BCA) method (Pierce BCA Protein Assay Kit, Thermo Fisher Scientific) following the manufacturer's instructions. Equal amounts of reduced proteins (15 μg) were separated on a 2-8% TRIS-acetate gel (Invitrogen) for large protein separation. Proteins were separated according to

their size under an electric current of 100 volts for 1 hour and 30 minutes and then transferred to a nitrocellulose membrane (Protan®, Whatman®, GE Healthcare, Versailles, France) for 1 hour and 20 minutes at 10 volts under liquid conditions. The transfer quality was systematically checked using Ponceau S staining. Membranes were incubated in a blocking buffer composed of 1X PBS and 5% milk for 1 hour with agitation. The primary antibodies rabbit anti-LRP2 (1:1000, Abcam, ab76969), was diluted in the working buffer (1X PBS - 0.5% milk - 0.1% Tween-20) and applied to the membrane, then incubated overnight at 4°C with agitation. After washing, the membranes were incubated with HRP-conjugated secondary antibody goat anti-rabbit, 1:5000, Vector Ref. PI-1000, London, UK) diluted in the same working buffer for 1 hour at room temperature. ECL Plus Western Blotting Detection Reagents (GE Healthcare, Orsay, France) were used for visualization. Mouse anti-GAPDH antibody (1:5000, Invitrogen Ref. AM4300) was used as an internal control. Bands were visualized using iBright Imaging Systems (Thermo Fisher Scientific, France) and quantified using ImageJ software (National Institutes of Health, University of Wisconsin, WI, USA). Statistical results were obtained using the non-parametric Mann-Whitney test (*$p<0.05$, **$p<0.01$, ***$p<0.001$) to evaluate significant differences. Statistical analysis was performed using GraphPad Prism 8 software (GraphPad Software, San Diego, CA). Original blots were displayed in Supplemental Data File 5.

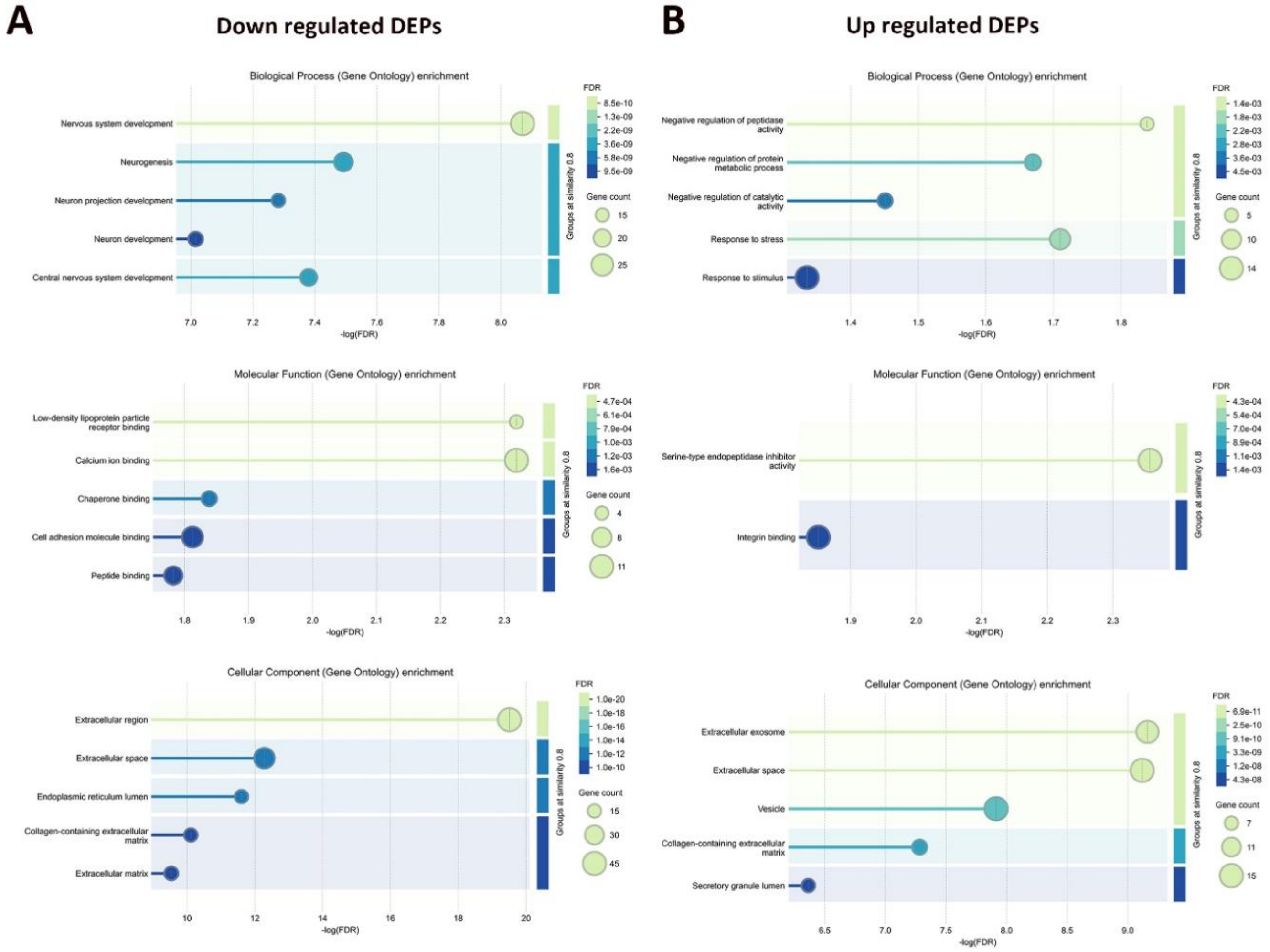

Supplementary Figure 1: GO analysis of the DEPs. (**A**) GO analysis of the down regulated proteins; (**B**) GO analysis of up regulated proteins. According to the order of FDR, only the top 5 terms were displayed. Gene count associated with a particular GO is indicated.

A

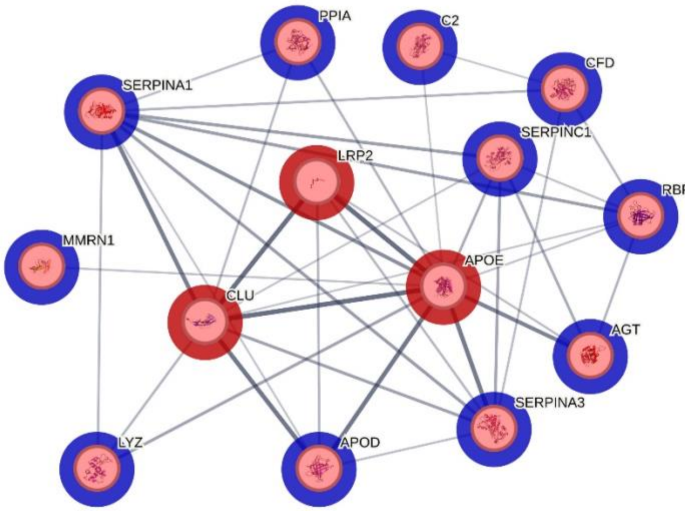

B

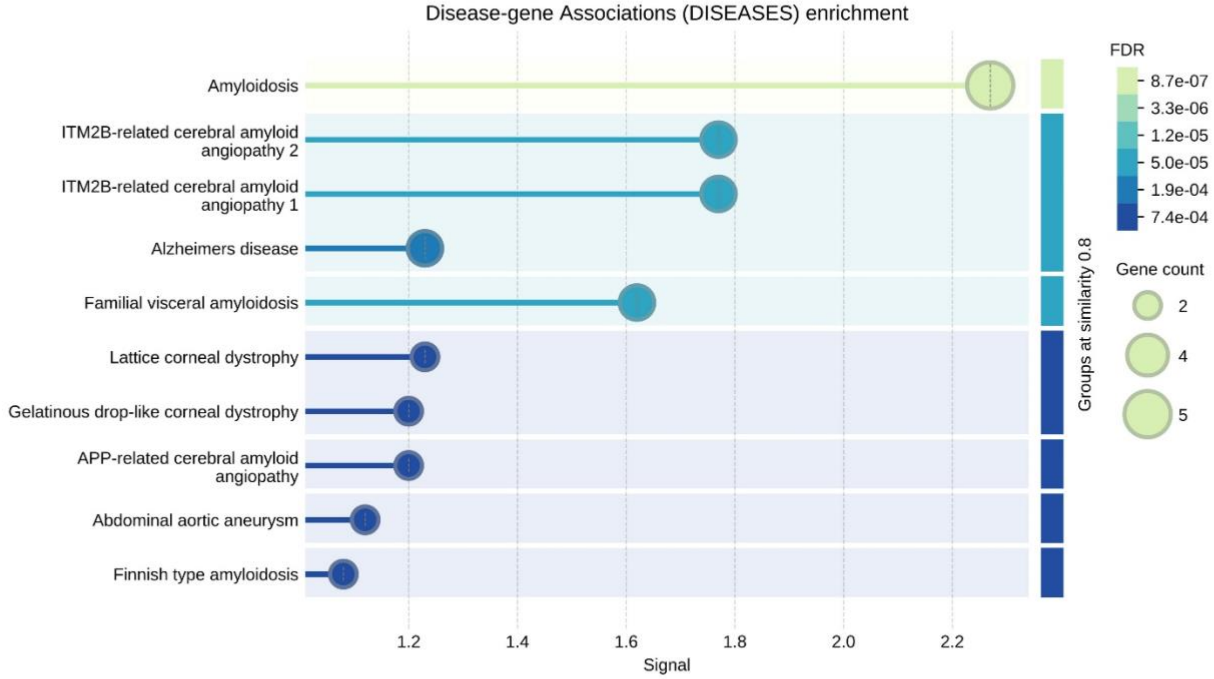

Supplementary Figure 2: (**A**) The most significant cluster was obtained from PPI network. LRP2 and its ligands CLU and APOE were enriched as hub proteins in this cluster. (**B**) Disease-gene associations enrichment indicated an enrichment in neurodegenerative diseases.

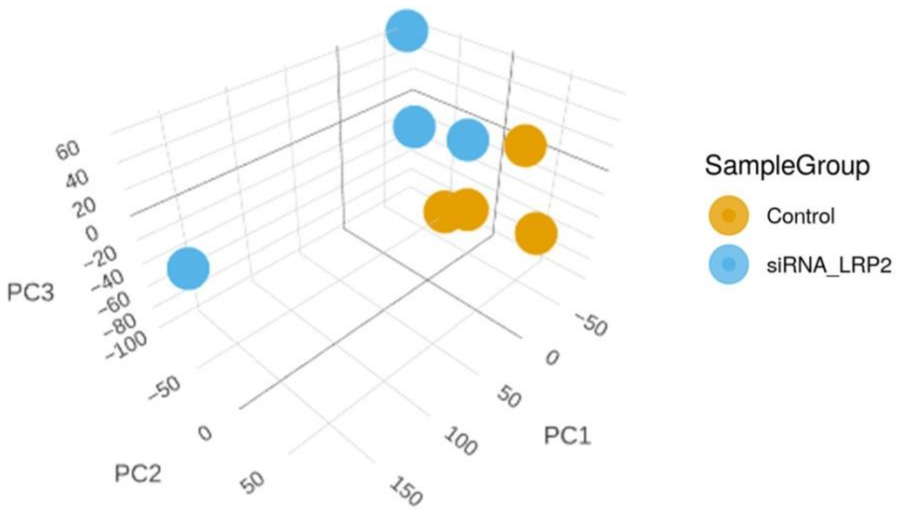

Supplementary Figure 3: Principal component (PC) analysis, X-axis, Y-axis, and Z-axis show PC1, PC2 and PC3, respectively.

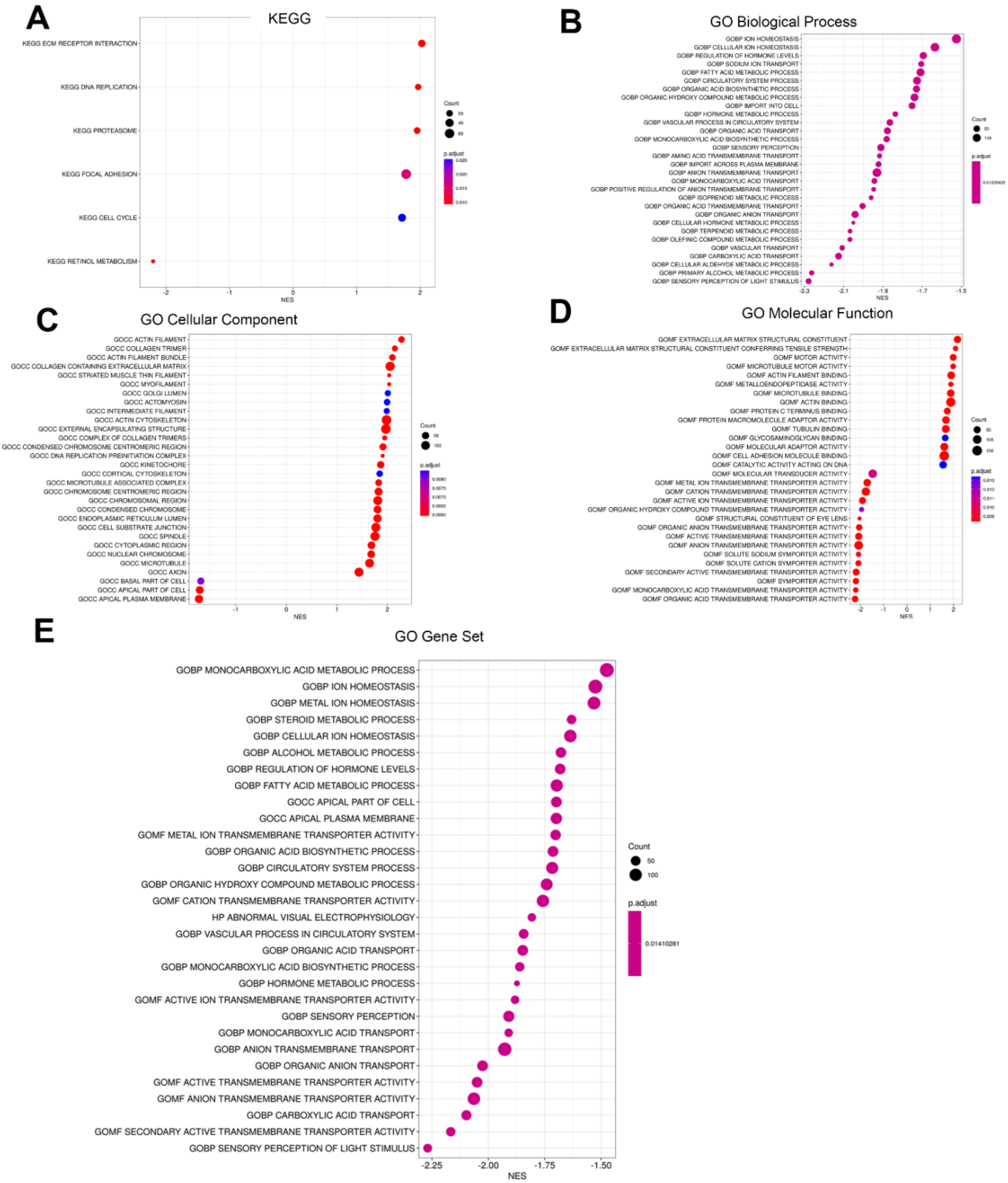
A
KEGG
KEGG ECM RECEPTOR INTERACTION
KEGG DNA REPLICATION
KEGG PROTEASOME
KEGG FOCAL ADHESION
KEGG CELL CYCLE
KEGG RETINOL METABOLISM
NES
Count
p.adjust
B
GO Biological Process
GOBP ION HOMEOSTASIS
GOBP CELLULAR ION HOMEOSTASIS
GOBP REGULATION OF HORMONE LEVELS
GOBP SODIUM ION TRANSPORT
GOBP FATTY ACID METABOLIC PROCESS
GOBP CIRCULATORY SYSTEM PROCESS
GOBP ORGANIC ACID BIOSYNTHETIC PROCESS
GOBP ORGANIC HYDROXY COMPOUND METABOLIC PROCESS
GOBP IMPORT INTO CELL
GOBP HORMONE METABOLIC PROCESS
GOBP VASCULAR PROCESS IN CIRCULATORY SYSTEM
GOBP ORGANIC ACID TRANSPORT
GOBP MONOCARBOXYLIC ACID BIOSYNTHETIC PROCESS
GOBP SENSORY PERCEPTION
GOBP AMINO ACID TRANSMEMBRANE TRANSPORT
GOBP IMPORT ACROSS PLASMA MEMBRANE
GOBP ANION TRANSMEMBRANE TRANSPORT
GOBP MONOCARBOXYLIC ACID TRANSPORT
GOBP POSITIVE REGULATION OF ANION TRANSMEMBRANE TRANSPORT
GOBP ISOPRENOID METABOLIC PROCESS
GOBP ORGANIC ACID TRANSMEMBRANE TRANSPORT
GOBP ORGANIC ANION TRANSPORT
GOBP CELLULAR HORMONE METABOLIC PROCESS
GOBP TERPENOID METABOLIC PROCESS
GOBP OLEFINIC COMPOUND METABOLIC PROCESS
GOBP VASCULAR TRANSPORT
GOBP CARBOXYLIC ACID TRANSPORT
GOBP CELLULAR ALDEHYDE METABOLIC PROCESS
GOBP PRIMARY ALCOHOL METABOLIC PROCESS
GOBP SENSORY PERCEPTION OF LIGHT STIMULUS
NES
Count
p.adjust
C
GO Cellular Component
GOCC ACTIN FILAMENT
GOCC COLLAGEN TRIMER
GOCC ACTIN FILAMENT BUNDLE
GOCC COLLAGEN CONTAINING EXTRACELLULAR MATRIX
GOCC STRIATED MUSCLE THIN FILAMENT
GOCC MYOFILAMENT
GOCC GOLGI LUMEN
GOCC ACTOMYOSIN
GOCC INTERMEDIATE FILAMENT
GOCC ACTIN CYTOSKELETON
GOCC EXTERNAL ENCAPSULATING STRUCTURE
GOCC COMPLEX OF COLLAGEN TRIMERS
GOCC CONDENSED CHROMOSOME CENTROMERIC REGION
GOCC DNA REPLICATION PREINITIATION COMPLEX
GOCC KINETOCHORE
GOCC CORTICAL CYTOSKELETON
GOCC MICROTUBULE ASSOCIATED COMPLEX
GOCC CHROMOSOME CENTROMERIC REGION
GOCC CHROMOSOMAL REGION
GOCC CONDENSED CHROMOSOME
GOCC ENDOPLASMIC RETICULUM LUMEN
GOCC CELL SUBSTRATE JUNCTION
GOCC SPINDLE
GOCC CYTOPLASMIC REGION
GOCC NUCLEAR CHROMOSOME
GOCC MICROTUBULE
GOCC AXON
GOCC BASAL PART OF CELL
GOCC APICAL PART OF CELL
GOCC APICAL PLASMA MEMBRANE
NES
Count
p.adjust
D
GO Molecular Function
GOMF EXTRACELLULAR MATRIX STRUCTURAL CONSTITUENT
GOMF EXTRACELLULAR MATRIX STRUCTURAL CONSTITUENT CONFERRING TENSILE STRENGTH
GOMF MOTOR ACTIVITY
GOMF MICROTUBULE MOTOR ACTIVITY
GOMF ACTIN FILAMENT BINDING
GOMF METALLOENDOPEPTIDASE ACTIVITY
GOMF MICROTUBULE BINDING
GOMF ACTIN BINDING
GOMF PROTEIN C TERMINUS BINDING
GOMF PROTEIN MACROMOLECULE ADAPTOR ACTIVITY
GOMF TUBULIN BINDING
GOMF GLYCOSAMINOGLYCAN BINDING
GOMF MOLECULAR ADAPTOR ACTIVITY
GOMF CELL ADHESION MOLECULE BINDING
GOMF CATALYTIC ACTIVITY ACTING ON DNA
GOMF MOLECULAR TRANSDUCER ACTIVITY
GOMF METAL ION TRANSMEMBRANE TRANSPORTER ACTIVITY
GOMF CATION TRANSMEMBRANE TRANSPORTER ACTIVITY
GOMF ACTIVE ION TRANSMEMBRANE TRANSPORTER ACTIVITY
GOMF ORGANIC HYDROXY COMPOUND TRANSMEMBRANE TRANSPORTER ACTIVITY
GOMF STRUCTURAL CONSTITUENT OF EYE LENS
GOMF ORGANIC ANION TRANSMEMBRANE TRANSPORTER ACTIVITY
GOMF ACTIVE TRANSMEMBRANE TRANSPORTER ACTIVITY
GOMF ANION TRANSMEMBRANE TRANSPORTER ACTIVITY
GOMF SOLUTE SODIUM SYMPORTER ACTIVITY
GOMF SOLUTE CATION SYMPORTER ACTIVITY
GOMF SECONDARY ACTIVE TRANSMEMBRANE TRANSPORTER ACTIVITY
GOMF SYMPORTER ACTIVITY
GOMF MONOCARBOXYLIC ACID TRANSMEMBRANE TRANSPORTER ACTIVITY
GOMF ORGANIC ACID TRANSMEMBRANE TRANSPORTER ACTIVITY
NES
Count
p.adjust
E
GO Gene Set
GOBP MONOCARBOXYLIC ACID METABOLIC PROCESS
GOBP ION HOMEOSTASIS
GOBP METAL ION HOMEOSTASIS
GOBP STEROID METABOLIC PROCESS
GOBP CELLULAR ION HOMEOSTASIS
GOBP ALCOHOL METABOLIC PROCESS
GOBP REGULATION OF HORMONE LEVELS
GOBP FATTY ACID METABOLIC PROCESS
GOCC APICAL PART OF CELL
GOCC APICAL PLASMA MEMBRANE
GOMF METAL ION TRANSMEMBRANE TRANSPORTER ACTIVITY
GOBP ORGANIC ACID BIOSYNTHETIC PROCESS
GOBP CIRCULATORY SYSTEM PROCESS
GOBP ORGANIC HYDROXY COMPOUND METABOLIC PROCESS
GOMF CATION TRANSMEMBRANE TRANSPORTER ACTIVITY
HP ABNORMAL VISUAL ELECTROPHYSIOLOGY
GOBP VASCULAR PROCESS IN CIRCULATORY SYSTEM
GOBP ORGANIC ACID TRANSPORT
GOBP MONOCARBOXYLIC ACID BIOSYNTHETIC PROCESS
GOBP HORMONE METABOLIC PROCESS
GOMF ACTIVE ION TRANSMEMBRANE TRANSPORTER ACTIVITY
GOBP SENSORY PERCEPTION
GOBP MONOCARBOXYLIC ACID TRANSPORT
GOBP ANION TRANSMEMBRANE TRANSPORT
GOBP ORGANIC ANION TRANSPORT
GOMF ACTIVE TRANSMEMBRANE TRANSPORTER ACTIVITY
GOMF ANION TRANSMEMBRANE TRANSPORTER ACTIVITY
GOBP CARBOXYLIC ACID TRANSPORT
GOMF SECONDARY ACTIVE TRANSMEMBRANE TRANSPORTER ACTIVITY
GOBP SENSORY PERCEPTION OF LIGHT STIMULUS
-2.25
-2.00
-1.75
-1.50
NES
Count
50
100
p.adjust
0.01410281

Supplementary Figure 4: (**A**) The top 6 most enriched KEGG of downregulated DEGs and upregulated DEGs. (**B to D**) The top 30 most enriched GO terms (selected based on the p-values), (**B**) GO biological processes, (**C**) GO cellular processes, (**D**) GO molecular functions, and (**E**) GO gene sets. The terms were selected based on the lowest LogP values (color codes on each graph). Analysis was performed using metascape. Absolute normalized enrichment allowed to identify terms that were upregulated or downregulated using all DEGs.

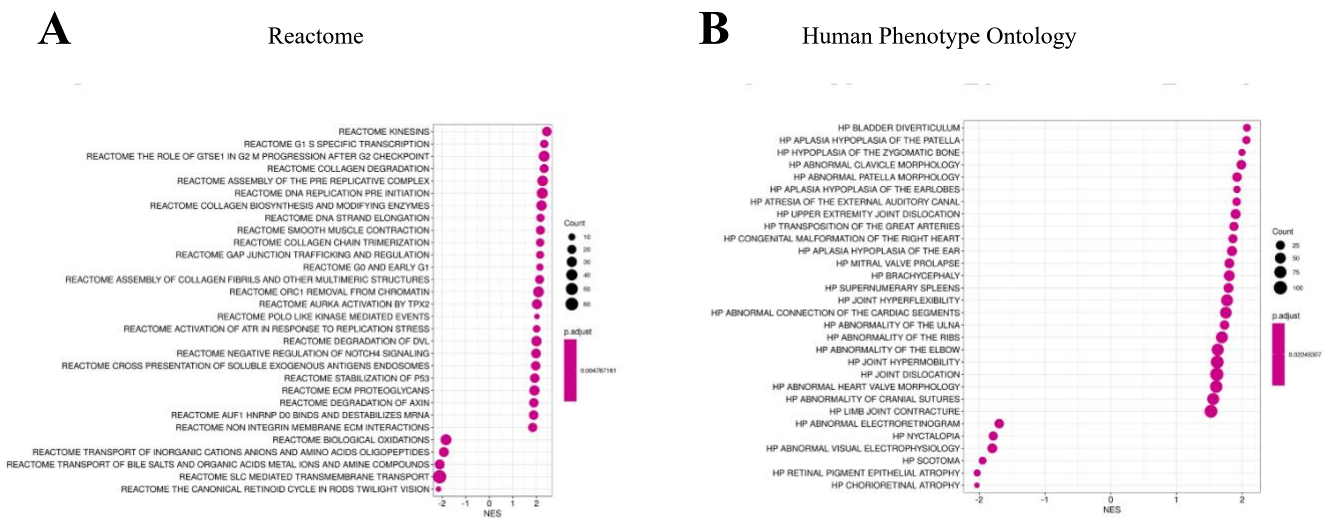

Supplementary Figure 5: The top 30 most enriched GO terms (selected based on the p-values), (**A**) reactome and (**B**) human phenotype ontology. The terms were selected based on the lowest LogP values (color codes on each graph). Analysis was performed using metascape. Absolute normalized enrichment allowed to identify terms that were upregulated or downregulated using all DEGs.

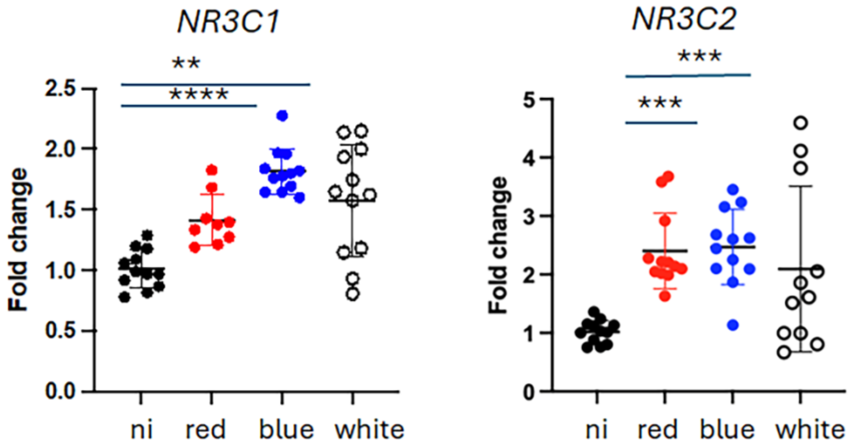

Supplementary Figure 6: Quantification of *NR3C1 and NR3C2* mRNA by Q-PCR in iRPE non-exposed (ne) or exposed to red, blue, or white light after 30 min (n=3), 2 hr (n=3) and 10 hr (n=3) after the last illumination. Time points (n=9) were grouped according to lighting conditions. Values correspond to the means of 4 independent experiments in duplicates for each condition. Each independent experiment represents the mean of three wells. Datas were expressed in fold gene expression ± SD. Data were analyzed using the non-parametric Kruskal Wallis test and Mann Witney post-test, $^{**}p<0.01$, $^{***}p=0.001$, $^{****}p<0.001$.

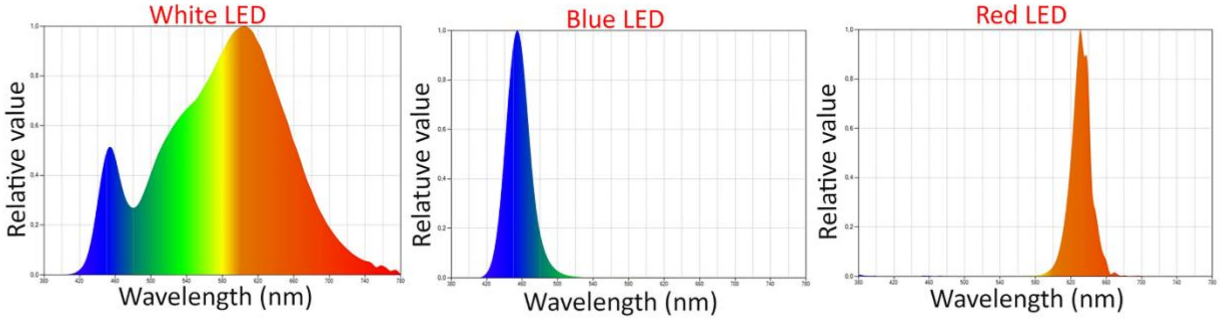

Supplementary Figure 7: Emission spectra of the LED lights used in this study.

REFERENCES

1. Cehofski LJ, et al. Aqueous Fibronectin Correlates With Severity of Macular Edema and Visual Acuity in Patients With Branch Retinal Vein Occlusion: A Proteome Study. *Invest Ophthalmol Vis Sci*. 2020;61(14):6.

2. Cehofski LJ, et al. IL-18 and S100A12 Are Upregulated in Experimental Central Retinal Vein Occlusion. *Int J Mol Sci*. 2018;19(11):3328.

3. Cehofski LJ, et al. Proteins involved in focal adhesion signaling pathways are differentially regulated in experimental branch retinal vein occlusion. *Exp Eye Res*. 2015;138:87–95.
