# Supplementary material for: LRP2 is a potential molecular target for nonsyndromic pathological myopia
Source: JCI Insight. 2025 Jun 24;10(15):e192929. doi: 10.1172/jci.insight.192929 (PMC12333944; doi:10.1172/jci.insight.192929)
Supplement: Unedited blot and gel images [file jciinsight-10-192929-s016.pdf]

Western blot of iRPE cells treated with shLRP2 :

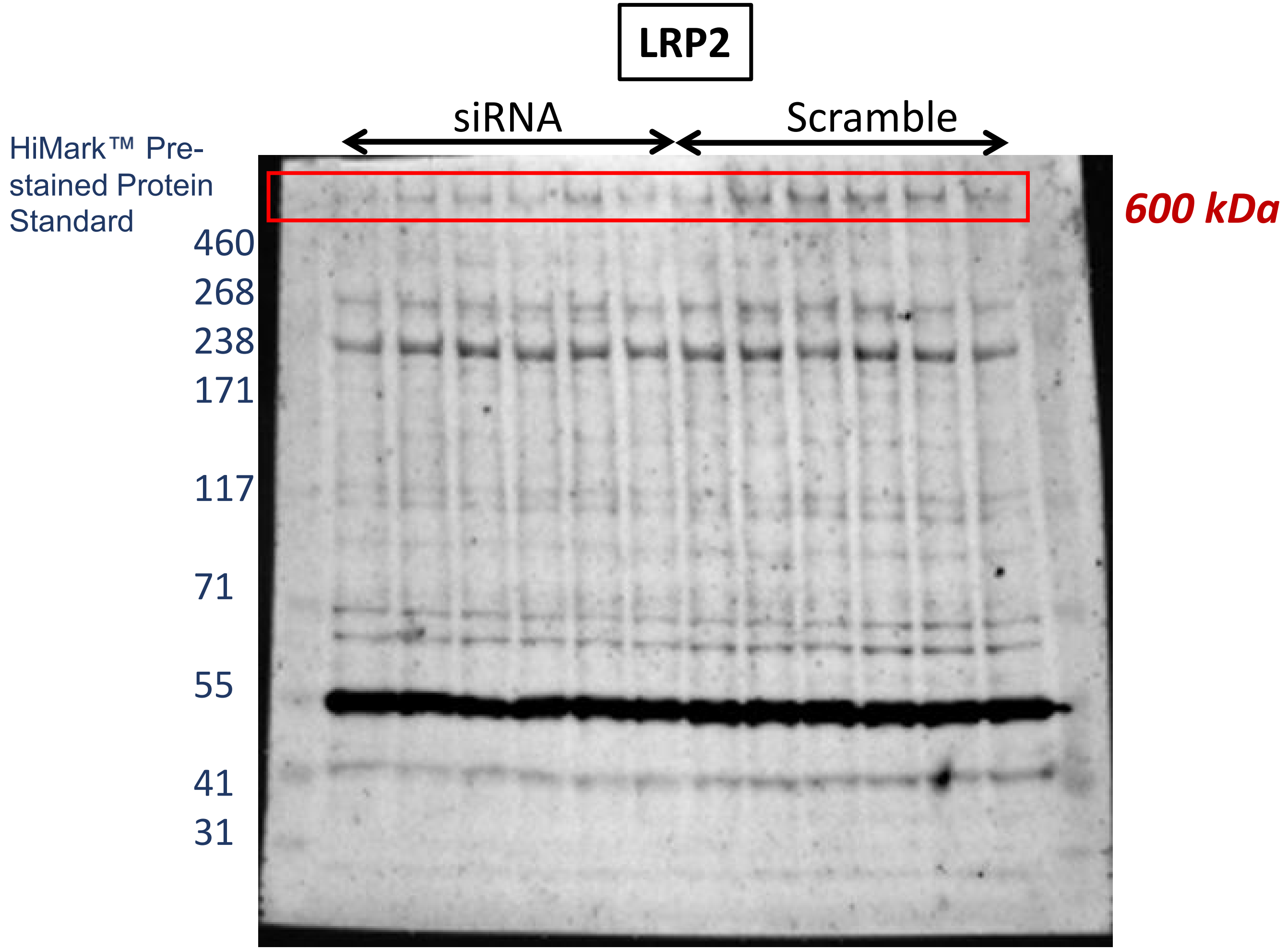

Gel 3-8% TRIS ACETATE

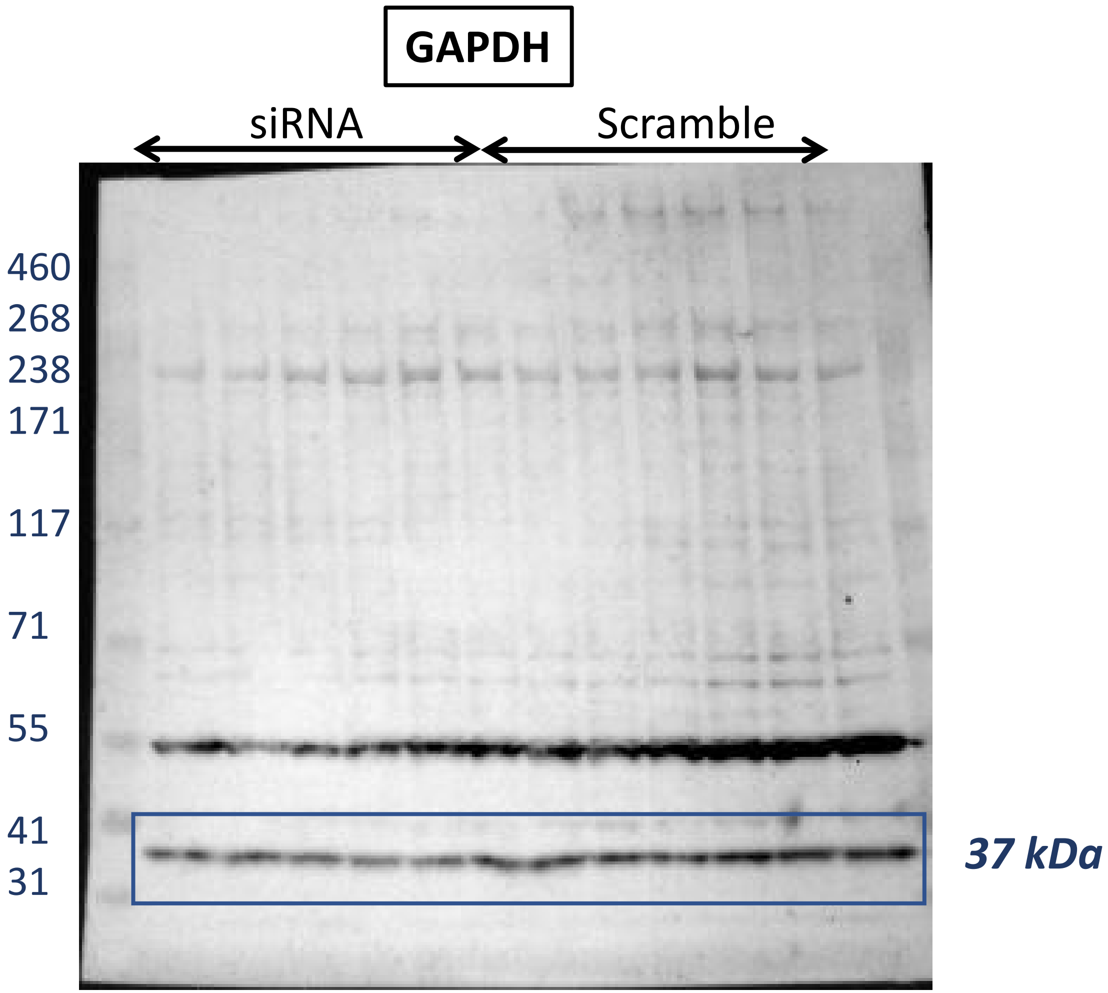

Image j quantification :

LRP2

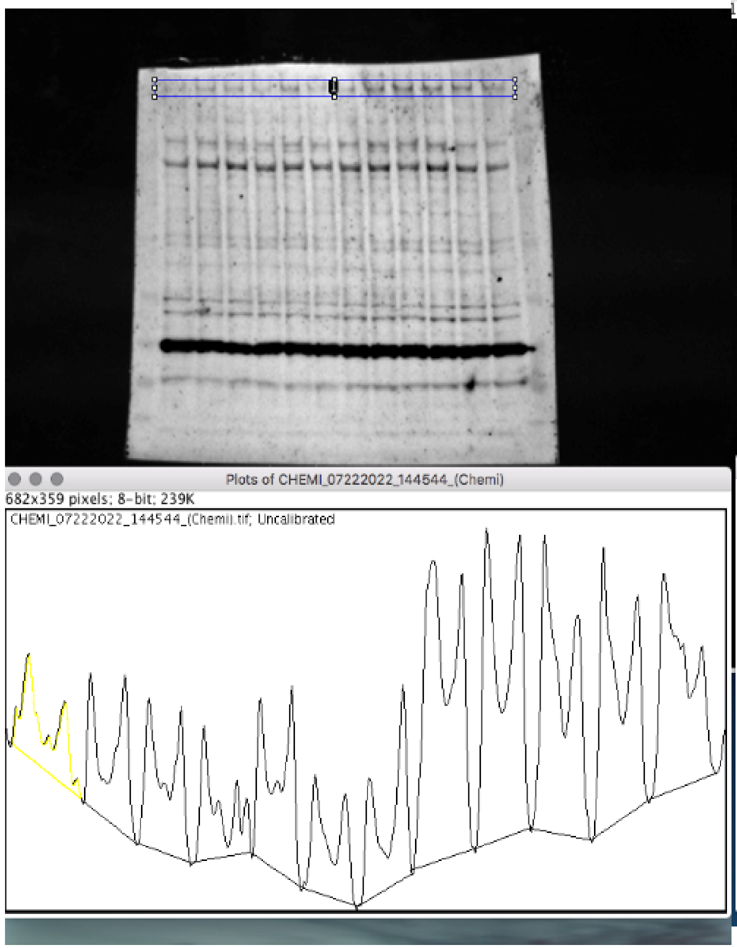

GAPDH

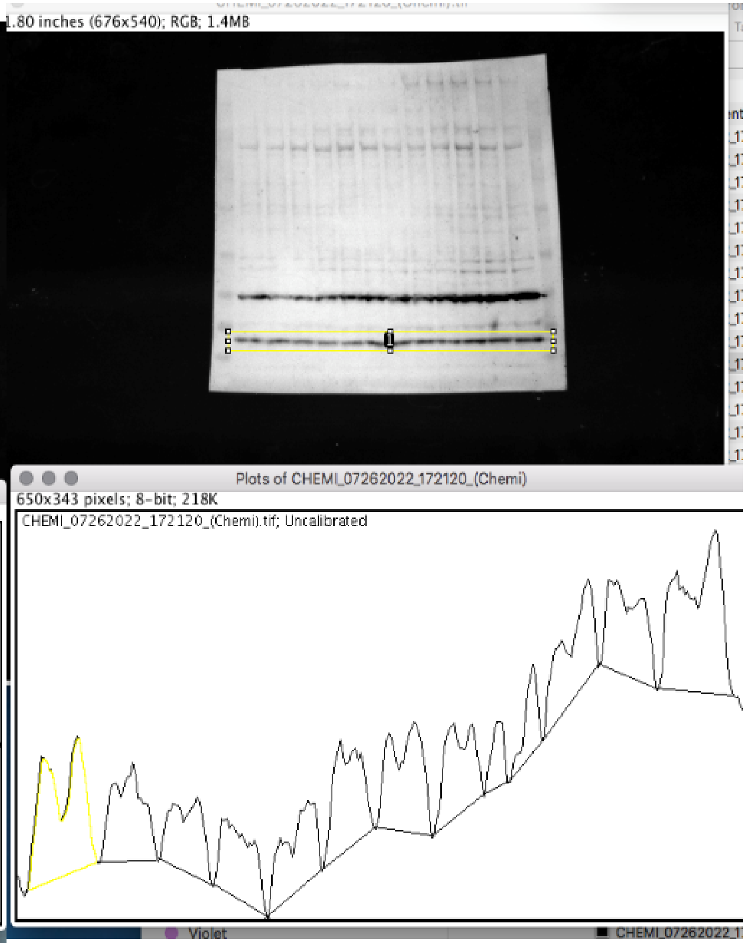

	Samples	LRP2 600 kDa	GAPDH 37 kDa	Report on GAPDH	Report on scramble average
siRNA	1	2182,7	4058,2	**0,54**	**0,19**
	2	2918,9	2293,6	**1,27**	**0,44**
	3	3274,7	1823,1	**1,80**	**0,62**
	4	2277,6	2029,5	**1,12**	**0,39**
	5	3698,3	1966,3	**1,88**	**0,65**
	6	2493,5	2303,9	**1,08**	**0,37**
**Average**				**1,28**	**0,44**
scramble	7	3154,2	2702,6	**1,17**	**0,40**
	8	8755,3	1889,3	**4,63**	**1,60**
	9	7697,0	1530,3	**5,03**	**1,73**
	10	7002,8	2366,2	**2,96**	**1,02**
	11	6117,8	2488,6	**2,46**	**0,85**
	12	5618,7	4858,1	**1,16**	**0,40**
**Average**				**2,90**	**1,00**
